# Programming bulk enzyme heterojunctions for biosensor development with tetrahedral DNA framework

**DOI:** 10.1038/s41467-020-14664-8

**Published:** 2020-02-11

**Authors:** Ping Song, Juwen Shen, Dekai Ye, Baijun Dong, Fei Wang, Hao Pei, Jianbang Wang, Jiye Shi, Lihua Wang, Wei Xue, Yiran Huang, Gang Huang, Xiaolei Zuo, Chunhai Fan

**Affiliations:** 1 Institute of Molecular Medicine, Department of Urology, Department of Nuclear Medicine, Renji Hospital, School of Medicine, Shanghai Jiao Tong University, 200127 Shanghai, China; 2 Frontiers Science Center for Transformative Molecules, School of Chemistry and Chemical Engineering, Shanghai Jiao Tong University, 200240 Shanghai, China; 3 Shanghai Key Laboratory of Regulatory Biology, Institute of Biomedical Sciences, School of Life Sciences, East China Normal University, 200241 Shanghai, China; 4 Division of Physical Biology, CAS Key Laboratory of Interfacial Physics and Technology, Shanghai Institute of Applied Physics, Chinese Academy of Sciences, 201800 Shanghai, China; 5 Shanghai Key Laboratory of Green Chemistry and Chemical Processes, School of Chemistry and Molecular Engineering, East China Normal University, 200241 Shanghai, China; 6 Bioimaging Center, Shanghai Synchrotron Radiation Facility, Zhangjiang Laboratory, Shanghai Advanced Research Institute, Chinese Academy of Sciences, 201210 Shanghai, China

**Keywords:** Bioanalytical chemistry, DNA nanotechnology

## Abstract

Protein-protein interactions are spatially regulated in living cells to realize high reaction efficiency, as seen in naturally existing electron-transfer chains. Nevertheless, arrangement of chemical/biochemical components at the artificial device interfaces does not possess the same level of control. Here we report a tetrahedral DNA framework-enabled bulk enzyme heterojunction (BEH) strategy to program the multi-enzyme catalytic cascade at the interface of electrochemical biosensors. The construction of interpenetrating network of BEH at the millimeter-scale electrode interface brings enzyme pairs within the critical coupling length (CCL) of ~10 nm, which in turn greatly improve the overall catalytic cascade efficiency by ~10-fold. We demonstrate the BEH generality with a range of enzyme pairs for electrochemically detecting clinically relevant molecular targets. As a proof of concept, a BEH-based sarcosine sensor enables single-step detection of the metabolic biomarker of sarcosine with ultrasensitivity, which hold the potential for precision diagnosis of early-stage prostate cancer.

## Abstract (web summary)

Tetrahedral DNA framework-enabled bulk enzyme heterojunctions have been used to program biosensor interfaces. Here, the authors use DNA tetrahedrons to tether enzymes of an enzymatic cascade to gold electrodes, hence raising them over the bulk solution, which led to improved kinetics and sensitivity.

## Introduction

Natually existing electron-transfer chains (ETCs) involve a series of spatially organized proteins, often localized in biological membranes, to harvest light or transduce energy with extraoridinarily high efficiency, as examplified by photosynthesis and cellular respiration^1,2^. As inspired, control on arranging chemical/biochemical components at the artificial device interfaces has led to the development of a wide range of artificial devices including solar cells^3,4^, fuel cells^5–7^, and biosensors^8,9^. Nevertheless, even when using natural proteins/enzymes in biomimetic devices, patterned assembly of proteins within the critical coupling length (CCL) for efficient reactions remains a grand challenge, which generally poses a limit in the device efficiency^10–12^. For example, whereas electrochemical biosensors hold great promise for portable and low-cost detection of clinically or environmentally relevant targets, glucose meters using single-component, glucose oxidase (GOX)-based electrodes are among the few biosensors with commercial success^13–16^. Biomimetical construction of multi-component enzyme electrodes for developing high-performance electrochemical biodevices has proven challenging due to the difficulty in organizing multi-enzyme-cascade within the CCL at the heterogeneous electrode interface^6,12^.

Structrual DNA nanotechnology represents a non-genetic approach to construct near-atomic biomolecular nanostructures^17–19^. Especially, DNA origami and other types of DNA nanostructures provide a general framework for organizing molecucles and materials in a plug-and-play manner with nanoscale addressability^19–28^. By exploiting the high programmability and precision of these nucleic acids framework, researchers have developed various protein assemblies to facilitate enzyme cascade, to replay intracellular signaling and to rewire molecular circuitry in vivo^11,12,29–31^. However, interfacing these solution-phase molecular assemblies to macroscopic biodevices has been rare, and replicating the nanoscale organization at the macroscopic scale has proven difficult^32,33^.

Tetrahedral DNA nanostructures (TDNs) are a type of three-dimensional nucleic acids framework with simple design, ordered structure and remarkable stiffness. More recently, they have been popularly employed to pattern biosensing interfaces, leading to uniform biorecognition layers for sensing a variety of biomolecular targets with remarkable sensitivity^8,34^. In addition to their demonstrated ability to engineer the biosensing interfaces, the intrinsic low cost of TDNs suits well with the biosensor development since they can be synthesized with only four designed DNA sequences and near-unity yield^8^. However, the use of TDNs has been restricted to pattern single-component biomolecules on electrodes due to the limited space available in these small-sized TDNs. Inspired by the donor-acceptor bulk heterojunction interface in semiconductor polymer solar cells^3,4^, here we develop a bulk enzyme heterojunction (BEH) strategy that employs TDNs to engineer the multi-enzyme-cascade electrode interface of biomimetic electrochemical biosensors. We show that patterning of the gold electrode with thiolated TDNs provides a uniform layer for near-homogeneous distribution of enzyme pairs within a CCL of ~10 nm. As a result, we observe high-efficiency multi-enzyme-cascade at the millimeter-scale electrode interface for highly sensitive detection of small-molecule targets. We further demonstrate that this BEH strategy enables ultrasensitive detection of sarcosine, a prostate cancer (PCa) biomarker for precision early-stage diagnosis and discrimination between benign prostatic hyperplasia (BPH) and PCa.

## Results

### The construction and characterization of BEH system

**Fig. 1:**
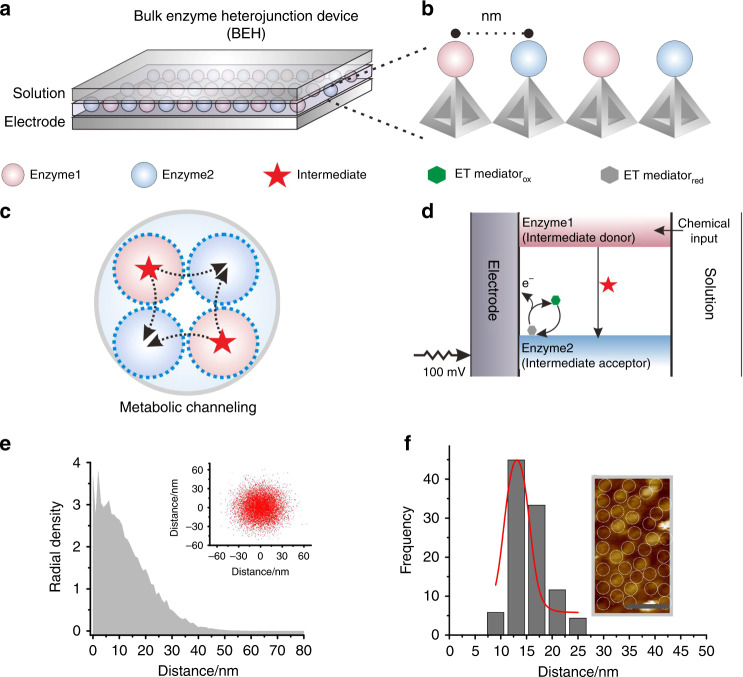
TDN-programmed BEH interface for biosensor development. **a** Schematic representation of the BEH strategy for enhanced the enzyme cascade reaction at the gold electrode interface. **b** The TDN scaffolds programmably regulate the inter-enzyme distance in BEH. **c** Substrate channeling in BEH that support efficient cascade among the enzyme pair (E1/E2) that are positioned with TDN scaffolds. **d** Working principle for BEH-based electrochemical biosensor device. The small molecule is oxidized by E1, producing an intermediate that diffuses to E2. As a result, electrocatalytic currents are observed at the electrode when potential is poised. **e** The radial density (molecule number in the area of 1 nm^2^) distribution of intermediate, as calculated from a random walk simulation. Inset: scatter chart of the distribution of intermediate. **f** Statistical results of the inter-enzyme distance of BEH, and corresponding AFM image of the enzymes at the interface. Scale bar: 50 nm. Source data are provided as a Source Data file.

The presented BEH system uses thiolated TDNs as the scaffold to spatially control the enzyme assembly at the interface of gold electrodes of 2-mm diameter. We first immobilized TDNs with three thiol-modified vertices (Supplementary Figs. 1–3, Supplementary Dataset 1 and Supplementary Table 1) on gold through the well-established Au-S chemistry^8,35^. In the resulting TDN layer, each TDN contains a top vertex pre-arranged with a unique pendant linker (UPL) DNA for site-specific anchoring of biomolecules, which enables the individual control of the enzyme organization (Supplementary Fig. 2). To construct a bienzyme electrode, we labeled the enzyme pair with two types of linker DNA, which were then hybridized in equimolar mixture to UPLs (UPL 1 and UPL 2) on the TDN-modified electrode (Fig. 1a, b and Supplementary Fig. 2). We reason that the inter-enzyme distance is defined by the size of TDNs that are well dispersed under Coulomb control; and that BEH enables optimized enzyme cascade within CCL. In a typical BEH device, in the presence of a target molecule (chemical input), the enzyme cascade reaction is initiated when an electronic input is applied to the electrode. The catalyzation of the target by the first enzyme (E1, e.g., sarcosine oxidase, SOX) concomitantly forms an intermediate, which serves as the substrate for second enzyme (E2, e.g., horseradish peroxidase, HRP). An electron-transfer mediator shuttles the electrons to the electrode to produce the electrochemical signal that translates to the target concentration (Fig. 1c, d).

We next employed redox labels (methylene blue (MB) or ferrocene  (Fc)) to quantify the amount of enzymes immobilized on the electrode surface^36–38^. Electrochemical characterization of redox labels tagged on DNA linkers revealed that the enzyme density was ~4.3 × 10^12^ cm^−2^ (Supplementary Fig. 4), which is comparable to the density of immobilized TDNs (~4.8 × 10^12^ cm^−2^). The inter-enzyme distance is then estimated to be ~5.5 nm, which is also comparable to the size of TDN (~5.7 nm). Atomic force microscopy (AFM) imaging of the enzyme-immobilized electrode demonstrated that the morphology of BEH is reasonably uniform, with an apparent inter-enzyme distance of ~15 nm (Fig. 1f and Supplementary Fig. 5). The larger value of the inter-enzyme distance observed under AFM might arise from the flexibility of chemical linkers between enzyme and TDN (Supplementary Fig. 6).

**Fig. 2:**
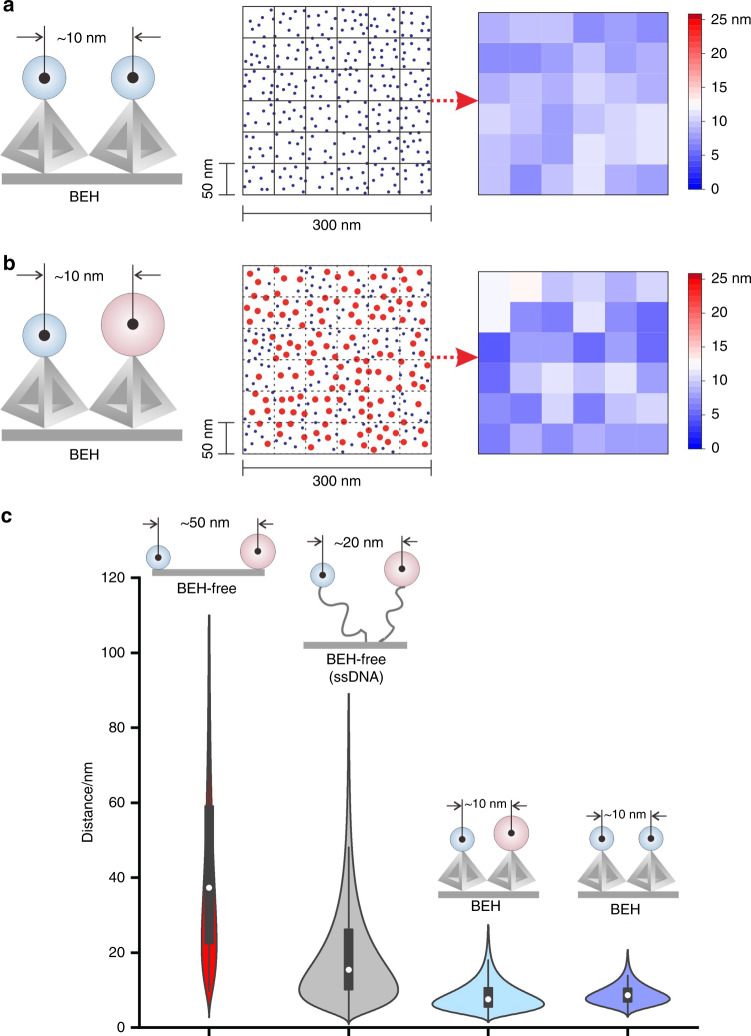
Comparison of the inter-particle distance-controllability. **a** The scheme and the locations (blue dots) of the assembled AuNPs (5 nm in diameter) on gold surface (from SEM image) by using BEH strategy. We measured the inter-particle distance of a representative region with 300 × 300 nm, by dividing the region into a 6 × 6 matrix. The heatmap demonstrated the average inter-particle distance of each square (50 × 50 nm region) in the matrix. The average inter-particle distance for the whole area is 9.1 ± 2.8 nm. **b** The scheme and the locations (blue and red dots) of the assembled AuNPs (5 nm and 10 nm in diameter) on gold surface (from SEM image) by using BEH strategy. Red dots indicate AuNPs of 10 nm; Blue dots indicate AuNPs of 5 nm. The inter-particle distance of AuNP hetero-pairs was measured. The inter-particle distance obtained by the BEH strategy with TDN of 17 bp was ~8.5 ± 3.8 nm. **c** The distribution of inter-particle distance obtained with BEH and BEH-free strategies (naked gold surface and ssDNA modified surface). The inter-particle distance obtained by BEH-free strategies (~49.7 ± 25.6 nm for naked gold surface and ~19.3 ± 12.2 nm for ssDNA modified surface) were significantly larger than 10 nm. In the boxplots the white dots represent the median value, the up and down bounds of box represent the first quartile and third quartile values and the vertical black line represent the whisker. The sample size used to derive statistics in **c** is 18, 118, 133, and 100, respectively. Source data are provided as a Source Data file.

The key development reported here is the ability to spatially control the distance between molecules or particles on gold surface by using the BEH strategy. Such spatial controllability can be measured using scanning electron microscope (SEM) when particles are appropriately arranged in close proximity to each other. We used BEH strategy to direct the assembly of two types of gold nanoparticles (AuNP-L_1_ and AuNP-L_2_) with 5-nm in diameter (which is comparable to the size of HRP and SOX enzymes). The SEM results demonstrated the morphology of AuNPs was uniform, with an inter-particle distance of ~9.1 nm (Fig. 2a and Supplementary Fig. 7). We further investigated the surface engineering capability of BEH to co-assemble two types AuNPs with different diameters (5 nm and 10 nm in diameter). The SEM results indicated the inter-particle distance was ~8.5 nm (Fig. 2b and Supplementary Figs. 8–10), which is consistent with that of the assembly of AuNPs of 5 nm indicating the controllability of BEH strategy is independent of the assembled entities. In addition, we compared the inter-particle distance obtained by BEH and BEH-free strategies (naked gold surface, and single-stranded DNA (ssDNA) modified gold surface) (Fig. 2c and Supplementary Figs. 10–12), we observed uniform distribution of inter-particle distance within 10 nm by using the BEH strategy. By contrast, the inter-particle distance was significantly larger (up to ~50 nm) and difficult to control using BEH-free strategies.

To explore the CCL-dependence of enzyme cascade, we used a two-dimensional rectangular DNA origami as a nanoscale ruler (Supplementary Figs. 13–20 and Supplementary Dataset 2). We characterized and verified the inter-enzyme distance between SOX and HRP using a combination of single-molecule technique including atomic force microscopy (AFM, Supplementary Figs. 21–26), total internal reflection fluorescence (TIRF, Supplementary Figs. 27, 28) and super-resolution stochastic optical reconstruction (STORM, Supplementary Figs. 29–31) microscopy. The distance between SOX and HRP was varied from 10 nm to 70 nm by prescribing UPLs on the origami (10, 30, 50, and 70 nm). We next examined the distance-dependent cascade efficiency of the origami nanoruler-confined enzyme pair. In a typical SOX-HRP cascade reaction, SOX catalyzes the oxidative demethylation of sarcosine, which produces hydrogen peroxide (H_2_O_2_) that forms the basis for HRP-mediated signal transduction. The enzyme cascade was visualized by the peroxidase-coupled color reaction with a chromogenic substrate-TMB (3,3′,5,5′-Tetramethylbenzidine) (Supplementary Figs. 32, 33). Interestingly, we found  that the reaction velocity was inversely proportional to the inter-enzyme distance of SOX and HRP (Supplementary Fig. 34). By considering the yield of the origami-enzyme complex^39^, we estimated that the CCL for the SOX-HRP pair was of 10 nm. The control of the inter-enzyme distance within 10 nm exhibited ~14-fold enhancement in reaction velocity as compared to free enzymes in solution (Supplementary Fig. 34), indicating the importance of CCL in enzyme cascade in addition to the free rotation of the “native-like” enzymes. When the conjugates were immobilized on the electrode surface, by precisely controlling the inter-enzyme distance with DNA origami, we could measure the distance-dependent cascade efficiency, which showed that the 10-nm inter-enzyme distance resulted in the highest efficiency (Supplementary Fig. 35).

We next interrogated the BEH effect on electrodes at the millimeter scale. Given that the inter-enzyme distance of BEH is within the CCL as measured on the DNA origami nanoruler (~10 nm), the enzyme cascade is expected to be efficient. We employed a 3D Brownian motion model to theoretically model the BEH system (Fig. 1e). In BEH, the intermediate diffuses out of the active center of E1 into the bulk solution, and then diffuses into the active center of E2, which are limited by mass transport efficiency (Fig. 1c, d). Our theoretical calculation revealed the radial density distribution of the intermediate H_2_O_2_ was distance-dependent, with ~81% of H_2_O_2_ localized in the <20 nm region, and drastically decreased availability of H_2_O_2_ beyond that distance (Fig. 1e). That is, H_2_O_2_ generated from the SOX can diffuse to the HRP with high efficiency within the BEH distance of <20 nm.

### Biosensor development for sarcosine

Next, we employed sarcosine assays to characterize the BEH-engineered electrode. In the presence of 0.1 mM sarcosine, we observed remarkable electrocatalytic signals, manifesting efficient enzyme cascade in BEH and electron relay to the electrode (Supplementary Fig. 36). Compared to a solution-phase assay (under the optimized conditions) of 0.1 mM sarcosine with freely diffusive SOX/HRP, we obtained a ~12.5-fold signal enhancement, owing to the significantly decreased inter-enzyme distance on BEH-engineered electrode (Supplementary Fig. 37). As a control, we also prepared electrodes with physically adsorbed SOX/HRP pairs with randomly distributed orientation, which resulted in ~3.2-fold signal enhancement (Supplementary Fig. 38). The hollow structure of TDN framework ensured the efficient electron transfer of electron-transfer mediator TMB (Supplementary Fig. 39). When we increased the distance by using TDN of 37 bp in edge length, we observed the suppressed catalytic signal compared to that of using TDN of 17 bp in edge length (Supplementary Fig. 40). Also of note, we found that a three-dimensional assembly of BEH exhibited similar catalytic efficiency with the two-dimensional assembly, which provide a means to increase enzyme loading, for further improving the sensing performance (Supplementary Fig. 41). When we used the designed TDN (37-17), which has equal height with TDN of 17 bp and larger edge length (37 bp in edge length), we found that electrode modified with TDN of 17 bp showed more than 2-fold higher signal than that of electrode modified with TDN (37-17), indicating the inter-enzyme distance is critical for the enzyme cascade on electrode surface (Supplementary Figs. 42–44).

### The generalizability of BEH system

**Fig. 3:**
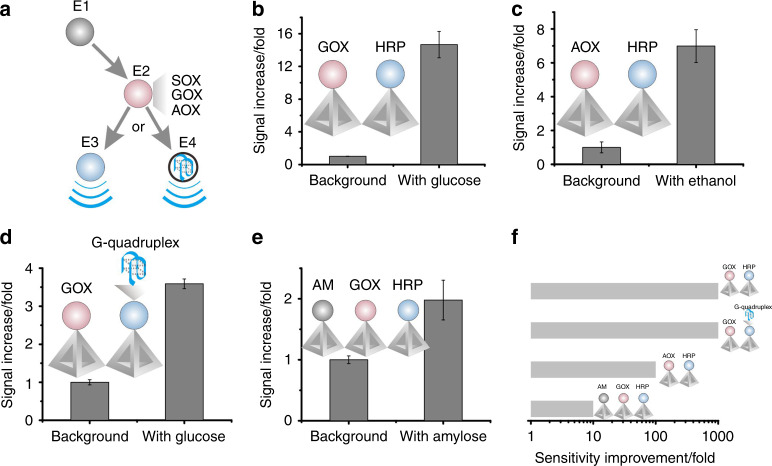
Generality of the BEH strategy for multi-enzyme-cascade on electrodes. **a** Design principle for bienzyme-cascade (E2/E3 or E2/E4) or trienzyme-cascade (E1/E2/E3) reactions. **b** Bienzyme-cascade for glucose detection using glucose oxidase (GOX) and HRP. **c** Bienzyme-cascade for ethanol detection using alcohol oxidase (AOX) and HRP. **d** Bienzyme-cascade for glucose detection using GOX and a DNAzyme (G-quadruplex-based peroxidase mimic). **e** Trienzyme-cascade for amylose detection using amyloglucosidase (AM), GOX and HRP cascade based BEH for the detection of amylose. **f** The sensors using BEH-engineered electrodes demonstrate sensitivity improvement by 1–3 orders of magnitude as compared to BEH-free ones. Error bars represent the mean ± s.d. of triplicates. Source data are provided as a Source Data file.

To demonstrate the generality of the design, we applied the BEH strategy to several multi-enzyme-cascade reactions (Fig. 3a). Similar to the SOX/HRP pair, the glucose oxidase (GOX)/HRP pair can be employed to construct a bienzyme-cascade BEH. The GOX-catalyzed oxidation of glucose produces intermediate H_2_O_2_ that serves as the substrate for HRP. In the presence of 1 mM glucose, we observed a signal gain of ~14.7, which compares favorably with ~1.7 gain for BEH-free electrodes (Fig. 3b, Supplementary Fig. 45). Analogously, we applied the BEH strategy to engineer two other bienzyme-cascade reactions, i.e., alcohol oxidase (AOX)/HRP for ethanol detection and GOX/DNAzyme for glucose detection, and a trienzyme-cascade reaction, i.e., amyloglucosidase (AM)/GOX/HRP for amylose detection. In all these BEH-engineered sensors, we observed remarkably enhanced signal gains, which directly translated to sensitivity improvement by 1–3 orders of magnitude (Fig. 3c–f, Supplementary Figs. 46–49).

### Sarcosine detection in biological relevant medium

**Fig. 4:**
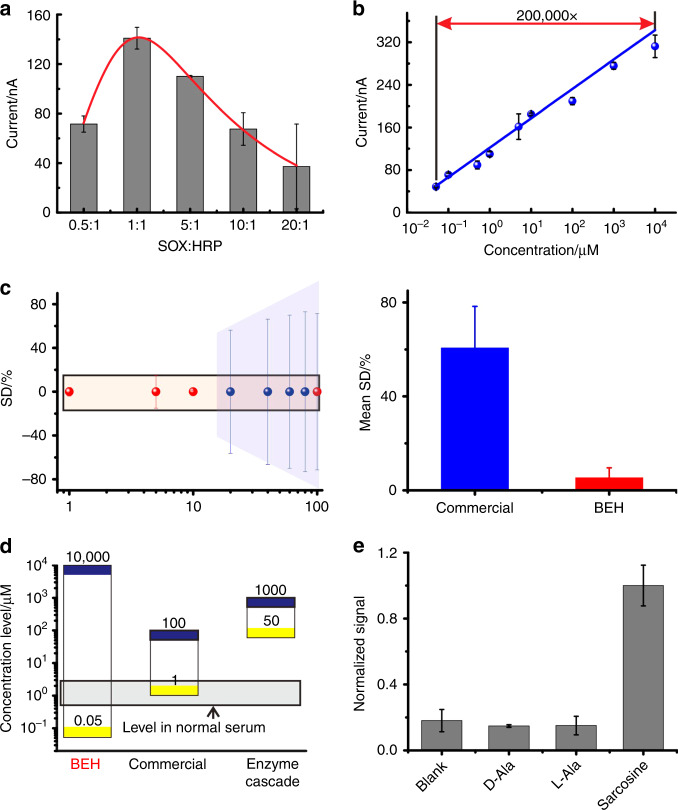
The sensing performances of BEH device. **a** The molar ratio of SOX and HRP was optimized. **b** The titration curve of sarcosine detection in serum, indicating the ability to detect sarcosine in complicated samples. The dynamic range spans from 50 nM to 10 mM. **c** Standard variation for a series of sarcosine dilutions for BEH and commercial kit (left). Mean standard variation for sarcosine dilutions for BEH and commercial kit. **d** Comparison of dynamic range of BEH, commercial kit, and enzyme cascade reaction in solution. The gray area indicates the sarcosine level in serum of healthy people. **e**The BEH device was highly specific to sarcosine. The signal from some other interferences such as the isomers of sarcosine (D-alanine and L-alanine) was similar to background signal. Error bars represent the mean ± s.d. of triplicates. Source data are provided as a Source Data file.

Having substantiated the BEH strategy at the biosensing interface, we explored the use of BEH-engineered biosensors for sarcosine assays in biologically relevant medium. We constructed BEH on a multiplexed 16-electrode chip (Supplementary Fig. 50) to electrochemically detect sarcosine in human serum. We found that the optimal BEH effect was achieved when the molar ratio of SOX/HRP was 1:1 (Fig. 4a). Electrochemical interrogation of sarcosine-spiked serum exhibited a linear dose-response curve spanning a broad dynamic range of over 5 orders of magnitude (Fig. 4b). The detection limit was estimated to be 50 nM (based on 3*δ* standard). Remarkably, compared to a commercial kit for sarcosine assays using the colorimetric or fluorometric method, our sensor excels in both the sensitivity and the dynamic range by two or three orders of magnitude, which allows direct detection of sarcosine at physiologically relevant concentrations (Fig. 4d, Supplementary Figs. 51, 52). Moreover, the BEH-engineered sensor exhibited significantly higher reproducibility than the commercial kit, as measured by the standard deviation across replicates (Fig. 4c, Supplementary Fig. 53). We also compared our sarcosine sensor with the high- performance liquid chromatography-mass spectra (HPLC-MS) method that has been the gold standard for sarcosine assays. Since our sensor obviates the additional derivatization step in HPLC-MS, it is a single-step assay for sensitive detection of sarcosine (50 nM) with the potential for high-throughput assays. (Supplementary Fig. 54).

To test the specificity of the BEH-engineered sensor, we challenged it with two isomers of sarcosine, i.e., D-Alanine and L-Alanine, which closely resemble sarcosine in the chemical structure. Importantly, the sensor signals for these two isomers were indistinguishable with the background (Fig. 4e), suggesting the high specificity of the BEH-engineered sensor. In contrast, the standard HPLC-MS  method for sarcosine assays does not possess the specificity to distinguish the isomers without the aid of additional derivatization step.

### The analysis of clinical samples

**Fig. 5:**
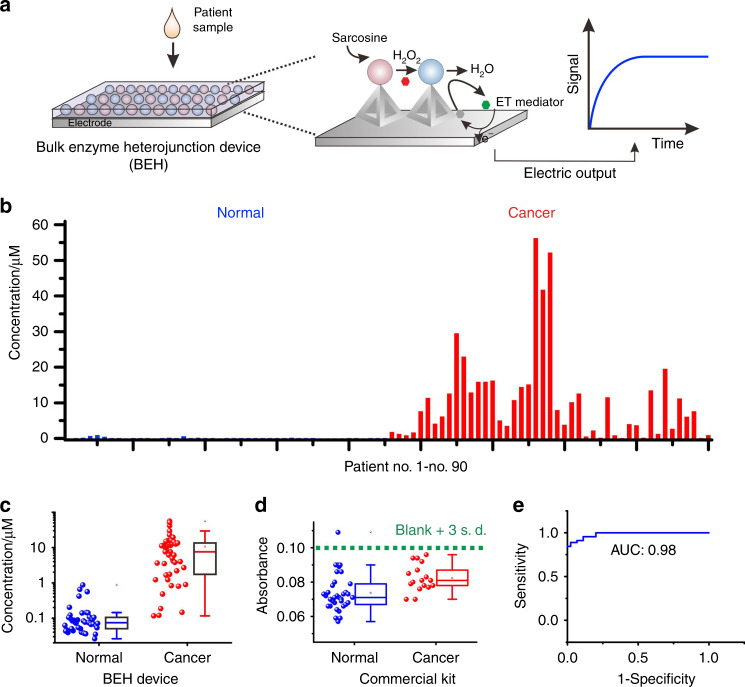
Sarcosine detection in clinical samples. **a** Schematics for the BEH-based sarcosine sensor. **b** Sarcosine detection with a multiplexed electrochemical chip in 90 clinical samples including serum from normal people (45 cases) and cancer patients (45 cases). **c** Statistical analysis reveals effective discrimination of cancer patients from normal people using the sarcosine sensor. **d** A control sarcosine assay with the commercial kit in clinical samples. Of note, most signals were in distinguishable from the background due to the poor sensitivity. **e** ROC analysis for the discrimination between normal and PCa using the sarcosine sensor. In the boxplots of **c** and **d** the horizontal line represent the median value, the up and down bounds of box represent the first quartile and third quartile values and the vertical lines represent the whisker. The sample size used to derive statistics is 45 for each group in **c**. The sample size used to derive statistics is 35 and 17 for groups in **d**. Source data are provided as a Source Data file.

Next, we employed the BEH-engineered sensor to detect sarcosine in real clinical samples (Fig. 5a). We collected serum samples from cancer patients (45 cases, Supplementary Dataset 3) and normal people (45 cases). By performing the sarcosine assays, we observed that the sarcosine level in the serum samples of cancer patients was significantly higher than that of normal controls (Fig. 5b, c). The receiver operating characteristic curve (ROC) indicated the high predictive power with the area under the curve (AUC) of 0.98 (Fig. 5e). When we defined the average sarcosine level of normal controls as the cutoff value, we obtained a sensitivity of 95.5% and specificity of 82.2%, respectively, which support the use of sarcosine as a metabolic biomarker for PCa. Interestingly, due to its low sensitivity, the commercial sarcosine kit could not effectively discriminate cancer patient samples from normal (Fig. 5d and Supplementary Fig. 55). Of note, the use of sarcosine as a metabolic biomarker raised arguments in previous reports; whereas the negative reports generally employed low-sensitivity methods (e.g., commercial kit)^40–46^. Our study thus partially explains the discrepancy, which is ascribed to the sensitivity for sarcosine assays.

Given that the discrimination of BPH from PCa is a major challenge in early-stage PCa detection, we challenged the sarcosine sensor in human serum samples from normal group (45 cases), BPH patients (45 cases), and cancer patients (45 cases). Importantly, the sarcosine detection exhibited significantly improved discrimination ability against BPH and PCa than the detection of popularly used PSA (Supplementary Figs. 56–58). Further analysis of these two biomarkers revealed that their detection had very low correlation coefficients (Supplementary Fig. 56). Given the orthogonality of the detection at protein and metabolic levels, we performed a combination assay using both PSA and sarcosine, which resulted in AUC of 1 for the discrimination of normal and PCa, and AUC of 0.75 for the discrimination of BPH and PCa (Supplementary Figs. 57, 58). The greatly improved discrimination ability of PSA/sarcosine sensing over the conventional PSA assays should facilitate the research on early-stage PCa diagnosis.

## Discussion

We have developed a TDN-programmed BEH strategy for engineering of the biosensing interface, which provides a generic approach for enhancing the enzyme cascade confined on the electrode surface. Although the control precision in the TDN-based system cannot be as high as that in the DNA origami system, i.e., the increased signal in the BEH-engineered system is due to an average of inter-enzyme distances on the electrode surface, this BEH strategy does provide a low-cost and highly generic approach to tune the cascade efficiency. The efficiency gain of the enzyme cascade can be directly translated to the sensitivity improvement for a broad range of multi-component enzyme biosensors, as exemplified by the success in fabricating a BEH-based sarcosine sensor. This sarcosine sensor can be directly deployed in complex biological fluids with high sensitivity, specificity and reproducibility, providing a low-cost, single-step and potentially high-throughput means for PCa screening. Given that both DNA- and enzyme- coated electrodes can be stored for months to even 1–2 years under appropriate conditions^39,47^, the BEH-engineered approach holds great promise for developing biosensors with long operation and storage stability.

## Methods

### Materials and reagents

Sarcosine (98%), tris(2-carboxyethyl) phosphine hydrochloride (TCEP), N,N-Dicyclohexylcarbodiimide (DCC), horseradish peroxidase (Type VI-A), glucose oxidase (*Aspergillus niger*), alcohol oxidase (*Pichia pastoris*), amyloglucosidase (*Aspergillus niger*), hemin, TCEP (Tris(2-carboxyethyl) phosphine), L-alanine, and D-alanine and sarcosine oxidase (*Bacillus sp.*) were purchased from Sigma and used without further purification. 3,3′,5,5′-tetramethylbenzidine (TMB) substrate, Alex 647 C2 maleimide and N-succinimidyl 3-(2-pyridyldithio)-propionate (SPDP) were purchased from Thermo scientific. Tris (hydroxy metheyl) (≥99%), boric acid (≥99.5%), magnesium acetate tetrahydrate (≥99%), amylose, glucose, ethanol and sodium hydroxide (≥96%) were purchased from Sinopharm Chemical Reagent Co. Ltd. M13mp18 DNA was purchased from Biolabs.

All solutions were prepared with Milli-Q water from a Millipore system.

The buffers used were as follows:

The TM buffer contains 20 mM Tris, 50 mM MgCl_2_, pH 8.0.

The PBS (pH = 7.0, 7.4 or 8.0) contains 12 mM phosphate buffer (PB), 2.7 mM KCl and 137 mM NaCl.

The hybridization buffer (pH 7.4) contains 10 mM phosphate buffer (PB), 20 mM MgCl_2_ and 1 M NaCl.

TAE-Mg^2+^ buffer (pH 7.5 or 8.0) contains 40 mM Tris, 20 mM acetic acid, 2 mM EDTA and 12.5 mM magnesium acetate.

DNA oligonucleotides were synthesized and purified by Sangon Biological Engineering Technology & Co. Ltd. (Shanghai, China) and stored in 1 × PBS. The sequences of these designed oligonucleotides were demonstrated in Supplementary Table 1, Supplementary Datasets 1 and 2.

### Protein-TDN conjugation

The protein and DNA were linked by SPDP^39^. The SOX and HRP were quantified with a Hitachi U-3010 UV−vis spectrophotometer (Japan). SOX was linked with linker 1 (L_1_), HRP was linked with linker 2 (L_2_), while amyloglucosidase was conjugated with Linker 3 (L_3_). DNA strands in Supplementary Table 1 were dissolved in H_2_O, yielding a final concentration of 50 μM. To fabricate TDNs, a quantity of 1 μL of each DNA strand as in Supplementary Datasets 1 was mixed with 5 μL of TCEP (30 mM) and 41 μL of TM buffer (20 mM Tris, 50 mM MgCl_2_, pH 8.0), and the resulting mixture was heated to 95 °C for 5 min, then cooled to 4 °C over 30 s, using a PeltierModel PTC-200 thermal cycler (MJ Research, Inc., SA). The final concentration of tetrahedron-structured probes (TDN) was 1 μM. Then the TDN1 (17TDN-1, 17/37TDN-1, 37TDN-1) was mixed with 3-fold of SOX-L_1_; TDN2 (17TDN-2, 17/37TDN-2, 37TDN-2) was mixed with 3-fold of HRP-L_2_; TDN3 (with strands A_3_BCD) was mixed with 3-fold of AOX-L_3_. The solution mixture was cooled from 37 °C to 4 °C with the following temperature gradient: 37 °C for 5 min; 36 °C-10 °C, 2 min per degree; 4 °C for storing the solution^39^.

### Gold electrodes processing

Gold electrodes were cleaned following the reported protocol^47^. The polycrystalline gold disk electrodes (2 mm diameter) were polished with a 300 nm and 50 nm alumina slurry on microcloth, respectively. And electrochemically cleaning by a series of oxidation and reduction cycling in 0.5 M NaOH (−0.4 to −1.35 V), 0.5 M H_2_SO_4_ (0–2 V), 0.5 M H_2_SO_4_ (0 to −0.35 V), 0.5 M H_2_SO_4_ (−0.35 to +1.5 V) and 0.01 M KCl/0.1 M H_2_SO_4_ (−0.2 to +1.25 V). Then 3 μL of TDN1-SOX and TDN2-HRP mixture with ratio of 1:1 was added to the cleaned gold electrode surface and allowed to react overnight at room temperature. The modified electrodes were rinsed with 0.01 M of phosphate-buffered saline (PBS) before electrochemical experiment.

### DNA origami preparation and conjugation with enzyme

DNA origami was prepared by using the same procedure with Rothemund’s^48^. M13mp18 DNA was chosen as the scaffold. Different staple DNAs were diluted in 200 nM for secondary structure. We mixed M13mp18 and different staple DNAs together in 1 × TAE-Mg^2+^ buffer with ratio of 1:10. The final concentration of M13mp18 was 5 nM. In order to link enzyme on the designed position of origami, we used our designed staple DNAs instead of the normal staples. For G1 (inter-enzyme distance of 10 nm), our designed staple DNAs were new-169, new-167, new-168 and new-166 instead of normal 169, 167, 168 and 166. For G3 (inter-enzyme distance of 30 nm), our designed staple DNAs were new-122, new-120, new-121 and new-119 instead of 122, 120, 121, and 119. For G5 (inter-enzyme distance of 50 nm), our designed staple DNAs were new-39, new-40, new-41, and new-42 instead of 39, 40, 41, and 42. For G7 (inter-enzyme distance of 70 nm), our designed staple DNAs were new 87, new 89, new 88, and new 90 instead of 87, 89, 88, and 90. To fabricate DNA origami (G1-SH, G3-SH, G5-SH, and G7-SH) for electrochemical analysis, we used our designed thiolated staple DNAs (SH1-SH12) instead of the normal staples (1, 2, 4, 20, 22, 24, 76, 77, 79, 93, 95, 97, 183, 185, 187, 201, 203, and 204) and TCEP was added in the mixed solution with final concentration of 3 mM. The mixed samples were annealed from 95 °C to 20 °C with steps of 0.1 °C per 10 s. The synthetic origami was centrifuge with 3500 × *g* for 10 min. The origami-enzymes conjugation was prepared by using the same method of TDN-enzyme conjugation. For origami-enzyme conjugation, LK-191 was linked with HRP and LK-167 was linked with SOX.

### Polyacrylamide gel analysis

Native 8% polyacrylamide gel (PAGE) analysis of the TDN: The DNA solution with 6 × loading buffer (TEK buffer, pH 8.0, 50% glycerol, 0.25% bromphenol blue) was analyzed in 8% native polyacrylamide gel. The electrophoresis was conducted in 1 × TBE (Mg^2+^ concentration: 12.5 mM; pH 8.0) at a constant voltage of 80 V for 2 h. The gels were scanned by a UV transilluminator after staining with Gel Red.

SDS-PAGE electrophoresis of protein-DNA conjugates: Conditions: spacer gel 5% and separation gel 12% with a constant voltage of 150 V for 70 min. All uncropped blots/gels are shown in Source Data.

### Electrochemical measurements

Electrochemical measurements were performed with a Model CHI 630b electrochemical workstation (CH Instruments, Inc., Austin, TX) and a conventional three-electrode configuration was employed throughout the experiment, which involved a gold working electrode, a platinum wire auxiliary electrode, and an Ag/AgCl (3 M KCl) reference electrode. Cyclic Voltammetry (CV) was carried out at a scan rate of 50 mV s^−1^. In addition, amperometric detection was fixed at 100 mV, and the electroreduction current was measured for 100 s after the HRP catalytic reaction reached the steady state. In order to achieve multiplexed detection, we used electrochemical 16-electrode arrays, which were provided by GeneFluidics (Monterey Park, CA). The serum was filtered by 10 kD filter before mixed in the TMB buffer. This process will not destroy the Sarcosine nor reduce the Sarcosine^44^. This work received ethical approval by Shanghai Jiao Tong University School of Medicine, Renji Hospital Ethics Committee. The patients’ samples were detected within 4 months after collection.

The surface area of gold electrode was determined by scanning CV in 0.05 M H_2_SO_4_. The charge (the area under the reduction peak) is converted to surface area using the parameter of 422 μC cm^−2^.

The total number of TDNs on electrode was determined by scanning ACV with MB or Fc modified TDNs as reported. The Alternating current voltammograms (ACV) was performed on the CHI630b electrochemical workstation using a three-electrode configuration. The Pt wire and Ag/AgCl (3 M KCl) were used as the counter electrode and reference electrode, respectively. The modified gold electrodes were thoroughly rinsed with 1 M NaClO_4_ and immersed in 1 M NaClO_4_ solution for electrochemical measurement. The ACV scanning were recorded from −0.5 V to 0.7 V. All experiments were conducted using a 25 mV AC potential at a frequency of 5 Hz.

TDNs surface density (N) was determined using a previously reported Eq. (1) (J. Electroanal. Chem. 1999, 466, 197–202):

1 $$I_{{{{\mathrm{avg}}}}}({{{\mathrm{E}}}}_0) = 2nfFN\frac{{{{{\mathrm{sinh}}}}(nFE_{{{{\mathrm{ac}}}}}/RT)}}{{\cosh \left( {\frac{{nFE_{{{{\mathrm{ac}}}}}}}{{RT}}} \right) + 1}}$$

where *I*_avg_(*E*_0_) is the average AC peak current, *n* is the number of electrons transferred per redox event (2 for MB and 1 for Fc), *F* is the Faraday constant, *R* is the universal gas constant, *T* is the temperature (Degree Kelvin), *E*_ac_ is the peak amplitude, and f is the frequency of the applied AC voltage perturbation.

### AFM characterization

For the characterization of origami-enzyme conjugations, 3 µL of sample was deposited onto a freshly cleaves mica surface and allowed to adsorb for 3 min. We added 300 µL of 1 × TAE-Mg^2+^ buffer to the liquid cell and the characterization was conducted using SNL-10 Tips (Bruker) by Multimode Nanoscope VII (Bruker) instrument in tapping mode.

For the characterization of TDN-enzyme conjugation, gold surface of electrochemical chip was cleaned with isopropanol by sonication for 2 min, and then cleaned in water for 20 min by sonication. The TDN was immobilized with the same method for electrochemical detection. The AFM characterization were conducted in peakforce mode.

### STORM and TIRF characterization

For STORM imaging, DNA linked HRP and SOX were labeled with Alexa Fluor 647 using Alexa Fluor 647 NHS Ester (Thermo Fisher) according to the protocol provided by the manufacturer. 20-fold of Alex 647 was mixed with LK-191 linked HRP and LK-167 linked SOX in 50 mM HEPES buffer (pH 8.5), respectively. The excess dyes were removed with 3-kD cutoff Amicon filters.

Samples were incubated on the surface of glass bottom cell culture dishes which are freshly treated by a plasma cleaner (Harrick Plasma PDC-32G cleaner). Super-resolution imaging experiments to characterize the inter-enzyme distance were performed using Nikon N-STROM system according to the manuals from Nikon. Briefly, STORM imaging buffer was prepared freshly on ice before each imaging experiment which contains 7 μL GOX solution (56 mg mL^−1^ Glucose Oxidase, 3.4 mg mL^−1^ Catalase, 10 mM Tris, 50 mM NaCl, pH 8.0), 70 μL of 1 M MEA and 620 μL of Buffer B (50 mM Tris, 10 mM NaCl, 10% Glucose, pH 8.0). We added 700 μL STORM imaging buffer into the dish and sealed the dished with parafilm. 100% of a 647 nm laser (200 mW) was used to record the binding events of imagers and the intensity of the 405 nm channel was adjusted to activate a proper number of dyes in dark state. The Andor DU-897 EMCCD readout bandwidth was set to 10 MHz at 14 bits and 5× conversion gain. EM gain was set to 100. Exposure time was chosen to be 100 ms to ensure a relatively high signal intensity for localization. In total we acquired 5000–30,000 frames for further analysis and super-resolution reconstruction. Super-resolution rendering was performed using the Nikon analysis software.

For resolution-limited co-localization of HRP and SOX with TIRFM, Alexa Fluor 647 NHS Ester was used to conjugate with HRP and Cy3 NHS Ester (Lumiprobe) was used to conjugate with SOX. 20-fold of Alexa Fluor 647 NHS Ester was mixed with LK-191 linked HRP and 20-fold of Cy3 NHS Ester was mixed with the LK-167 linked SOX in 50 mM HEPES buffer (pH 8.5). The excess dyes were removed with 3-kD cutoff Amicon filters.

We used the TIRF mode of the N-STORM system to image the fluorescence signals of HRP and SOX with 561 nm and 647 nm excitation lasers. The exposure time is 200 ms, and the laser intensity was adjusted to rule out inter-channel crosstalk.

Our STORM based inter-enzyme distance measurement method was based on the reported method (Mortensen, K. I.; Churchman, L. S.; Spudich, J. A.; Flyvbjerg, H. Optimized localization analysis for single-molecule tracking and super-resolution microscopy. Nat. Methods 2010, 7, 377.) with detailed theoretical and experimental analysis. The image analysis was conducted with Nikon N-STROM system according to the manuals from Nikon. In brief, we used fluorescent beads as fiducial markers and obtained over thousands of time-lapse frames. After peak height filtering, drift correction and other correction processes, the localization precision of our measurement was improved to ~10 nm, which could be applied to identify the inter-enzyme distance of 30–70 nm.

### Enzyme cascade assays in solution

The enzyme cascade assays of free enzymes (SOX and HRP) were conducted with multimode microplate reader (Synergy H1, BioTek). 1 mM of ABTS was added for monitoring the absorbance change at 410 nm and the reaction buffer was PBS. The final concentration of SOX and HRP and ratio was improved as in Supplementary Fig. 33.

The enzyme cascade assays of origami-enzyme conjugations were conducted with HITACHI U-3010 spectrophotometer by monitoring the increase of TMB in absorbance at 370 nm. The TMB solution was dilute by 4.5-fold, and the reaction buffer was TM buffer (pH: 8.0). The final concentration of origami-enzyme conjugation was 2.5 nM, the final sarcosine was 10 mM in 1× PBS buffer. The detections of sarcosine with sarcosine assay kit (Biovision, Sarcosine colorimetric/fluorometric assay kit, Catalog #K636-100) were conducted following the protocol of the commercial sarcosine assay kit.

### HPLC-MS detection of sarcosine

Before HPLC detection, we used N,N-Dicyclohexylcarbodiimide (DCC) as a derivatization reagent for sarcosine, D-alanine and L-alanine^49^. High-performance liquid chromatography-mass (HPLC–MS) experiment was conducted with HPLC analyzer (Agilent 6410 series Triple Quad). The analytical column was Agilent C18 column, maintained at 30 °C. The injection volume was 10 μL. A microsplitter valve (Agilent Technologies, Palo Alto, CA) delivered 50% of the flow to the mass spectrometer. Capillary voltage was set at 3500 V. High purity nitrogen (99.999%) was used as collision gas. Chromatograms were obtained in the positive ion and multiple reactions monitoring mode (ESI^+^-MRM). Detections were performed in MRM mode at *m/z* 296 for the derivatives of sarcosine, L-alanine, and D-alanine and at *m/z* 114 for creatinine.

### Random walk simulation

We used random walk model to animate the trajectory of a particle moving (http://www.excelunusual.com/tag/monte-carlo-modeling/). In this model, for each time step, a single H_2_O_2_ particle moves a fixed length L, at a randomly chosen angle *θ*. we wanted to know the distribution of the particles after many steps had been taken. Based on the situation of particle diffusion in fluids, we chose the free random walk model, which means the angle of movement *θ* is continuously and uniformly distributed between zero to 360 degrees with respect to the x axis. Another parameter for random walk is step size L, which was chosen as 0.287 nm. This value corresponding the outer diameter of H_2_O_2_.

Therefore, for any single particle, the position at t + 1 timepoint can be iteratively calculated as Eqs. 2 and 3:

2 $$x_{{{{\mathrm{t}}}} + 1} = x_{{{\mathrm{t}}}} + 0.287 \ast {{{\mathrm{cos}}}}\theta$$

3 $$y_{{{{\mathrm{t}}}} + 1} = y_{{{\mathrm{t}}}} + 0.287 \ast {{{\mathrm{sin}}}}\theta$$

In order to show it in statistical way, we mimicked 10,000 independent particles with a free rule of random walk movement at the same time. As soon as the first particle reached the range of 70 nm, which is the largest distance of our origami design, the model fixed. Here the final model stopped after N = 12,670 steps of length L = 0.287. The final positions of 10,000 independent Pearson random walks released from the origin were shown in Fig. 1e.

The area value is square growth with the radius *r*, in order to compare across bin easier, the density of each radius was calculated with Eq. 4 by division of the current number of particles contained within a circle of a certain radius centered and the area of this region.

4 $$\rho _1 = \frac{{N_1}}{{\pi \times r_1^2}} , \, \rho _2 = \frac{{N_2 - N_1}}{{\pi \times (r_2^2 - r_1^2)}}, \ldots \ldots \rho _{70} = \frac{{N_{70} - N_{69}}}{{\pi \times (r_{70}^2 - r_{69}^2)}}$$

Here *N*_1_, *N*_2_……*N*_70_ is the number of particles within a circle of a certain radius *r* (*r*_1_ = 1, *r*_2_ = 2… …*r*_70_ = 70).

### Preparation of DNA modified gold nanoparticles

The preparation of DNA modified gold nanoparticles (AuNPs) was according to reported methods (J. Am. Chem. Soc. 2012,134, 7266-7269). The AuNPs were incubated with 9.2 mM of BSPP for 1 h and further concentrated in 2.5 mM BSPP. The final concentrations of AuNPs of 5 nm and 10 nm in diameter were 500 nM and 200 nM, respectively. Add DNA solution (L_1_ or L_2_) into the concentrated AuNPs suspension with DNA to Au molar ratio of 50:1 (5 nm AuNPs) and 100:1 (10 nm AuNPs), respectively. Then citrate buffer (pH 3.0) was added into the mixed system to change the pH of finally system to 3.5. The mixtures were shaken with 350 rpm at 25 °C for 2 h and centrifuge the mixture for 20 min at 25 °C of 16,200 × *g*. Discard supernatants and resuspend precipitates with PBS. Repeat the resuspension for three times and make the finally concentration of AuNPs suspension to 1 μM. Store the AuNPs suspension at 4 °C before further experiments.

### Assembly of AuNPs on gold surface

Gold layer (50 nm thickness) coated on silicon wafer was used as gold plane surface for AuNPs assembly. The gold coated silicon wafer was sonicated in ethanol and water for 3 min, respectively and dried with N_2_ flow. The DNA probe (ssDNA, 17 bp TDNs and 37 bp TDNs) solution was prepared and dropped on the cleaned gold surface and incubated overnight at room temperature for DNA probes assembly. The AuNPs suspension of different size and modification were mixed at equal proportion as required. The final concentration of AuNPs in mixture was controlled as 1 μM. After rinsing with PBS dried under N_2_ flow, the gold coated silicon wafer was incubated with DNA modified AuNPs mixture (L_1_-5 nm AuNPs, L_2_-5 nm AuNPs, L_1_-5 nm AuNPs and L_2_-5 nm AuNPs, L_1_-5 nm AuNPs, and L_2_-10 nm AuNPs) at 37 °C for 12 h. After incubation, the gold coated silicon wafer was rinsed with PBS to remove unbound AuNPs and desalted with water rinsing. The gold coated silicon wafer was dried under infrared lamp and stored before SEM characterization.

### SEM characterization

The prepared gold coated silicon wafers with AuNPs assembled were directly characterized with SEM (1530VP; Zeiss Leo).

### BEH-free method

Seven microliter of mixed enzyme solution with the same enzyme concentration used in BEH experiments (3 μM for each enzyme and G-Quadruplex/hemin) was dropped on carbon electrode (diameter of 5 mm). The electrodes were kept at 4 °C overnight to dry. For each group of electrochemical detection, the conditions were controlled the same with that in BEH experiments. To compare the detection ability between experiment group (BEH) and the control group (BEH-free), we compared their detection sensitivity through 3*δ* standard and calculated the folds of sensitivity improvement.

### Reporting summary

Further information on research design is available in the Nature Research Reporting Summary linked to this article.

## Supplementary information

Supplementary Information

Peer Review File

Description of Additional Supplementary Files

Supplementary Data 1

Supplementary Data 2

Supplementary Data 3

Reporting Summary

## Data Availability

The main data supporting the findings of this study are available within the article and its Supplementary Information files. Extra data are available from the corresponding author upon reasonable request. The source data underlying Figs. 1e, f, 2c, 3d–f, 4, 5b–e, and Supplementary Figs. 3, 5, 20, 22, 31, 32c, d, 33, 34, 35b, 36, 38, 40d, e, 41c, 42d, 43e, 44, 45, 46–49, 51–53, 54b, 55–57 are provided as a Source Data file.

## Source Data

Source Data
