# Author Correction: Programming bulk enzyme heterojunctions for biosensor development with tetrahedral DNA framework

**DOI:** 10.1038/s41467-022-29628-3

**Published:** 2022-04-04

**Authors:** Ping Song, Juwen Shen, Dekai Ye, Baijun Dong, Fei Wang, Hao Pei, Jianbang Wang, Jiye Shi, Lihua Wang, Wei Xue, Yiran Huang, Gang Huang, Xiaolei Zuo, Chunhai Fan

**Affiliations:** 1 Institute of Molecular Medicine, Department of Urology, Department of Nuclear Medicine, Renji Hospital, School of Medicine, Shanghai Jiao Tong University, 200127 Shanghai, China; 2 Frontiers Science Center for Transformative Molecules, School of Chemistry and Chemical Engineering, Shanghai Jiao Tong University, 200240 Shanghai, China; 3 Shanghai Key Laboratory of Regulatory Biology, Institute of Biomedical Sciences, School of Life Sciences, East China Normal University, 200241 Shanghai, China; 4 Division of Physical Biology, CAS Key Laboratory of Interfacial Physics and Technology, Shanghai Institute of Applied Physics, Chinese Academy of Sciences, 201800 Shanghai, China; 5 Shanghai Key Laboratory of Green Chemistry and Chemical Processes, School of Chemistry and Molecular Engineering, East China Normal University, 200241 Shanghai, China; 6 Bioimaging Center, Shanghai Synchrotron Radiation Facility, Zhangjiang Laboratory, Shanghai Advanced Research Institute, Chinese Academy of Sciences, 201210 Shanghai, China

**Keywords:** Bioanalytical chemistry, DNA nanotechnology

Correction to: *Nature Communications* 10.1038/s41467-020-14664-8, published online 11 February 2020.

The original version of the Supplementary Information associated with this Article contained an error in Supplementary Figure 24, in which the wrong image of DNA origami was inadvertently used in the middle panel. The original version of the Supplementary Information associated with this Article contained an error in the caption of Supplementary Figure 24, which incorrectly read ‘The sample size used to derive statistics is 558’. The correct version states ‘The sample size used to derive statistics is 538’ in place of ‘The sample size used to derive statistics is 558’. The HTML has been updated to include a corrected version of the Supplementary Information.

## Supplementary information

Corrected Supplementary Information

