# Supplementary material for: Programming bulk enzyme heterojunctions for biosensor development with tetrahedral DNA framework
Source: Nat Commun. 2020 Feb 11;11:838. doi: 10.1038/s41467-020-14664-8 (PMC7012893; doi:10.1038/s41467-020-14664-8)
Supplement: Supplementary file 1 — Supplementary Information [file 41467_2020_14664_MOESM1_ESM.pdf]

Supplementary Information

Programming bulk enzyme heterojunctions for biosensor development with tetrahedral DNA framework

Song, Shen, Ye & Dong et al.

This PDF file includes:

Supplementary Figures 1 to 58

Supplementary Tables

Supplementary Notes

Supplementary References

Suplementary Figures

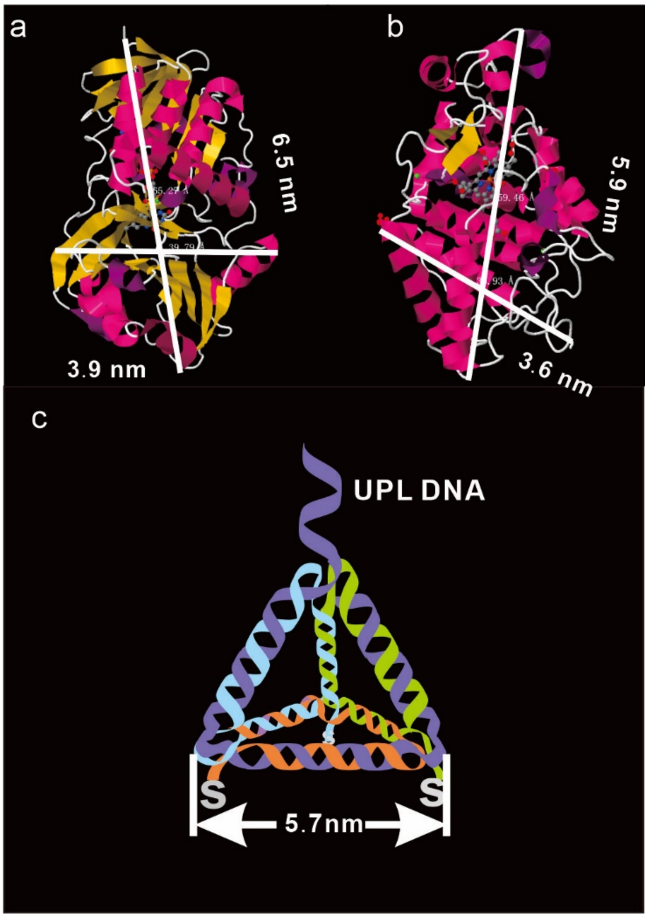

Supplementary Figure 1. Crystal structures of enzymes from Protein Data Bank (https://www.rcsb.org/). (a) sarcosine oxidase (SOX, PDB code 3QSE), (b) horseradish peroxidase (HRP, PDB code 1GWU). (c) Structure of DNA tetrahedron.

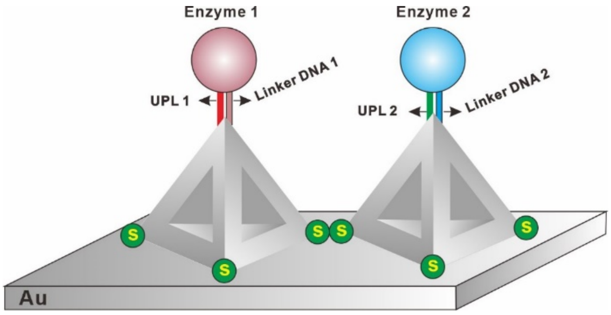

Supplementary Figure 2. Scheme of TDN immobilization on gold electrode. Three thiolated-DNAs on the three vertices at bottom was used to anchor TDN on gold surface. Each TDN contains a top vertex pre-arranged with a unique pendant linker (UPL) DNA for site-specific anchoring of enzyme 1 and enzyme 2, which enables the individual control of the enzyme organization.

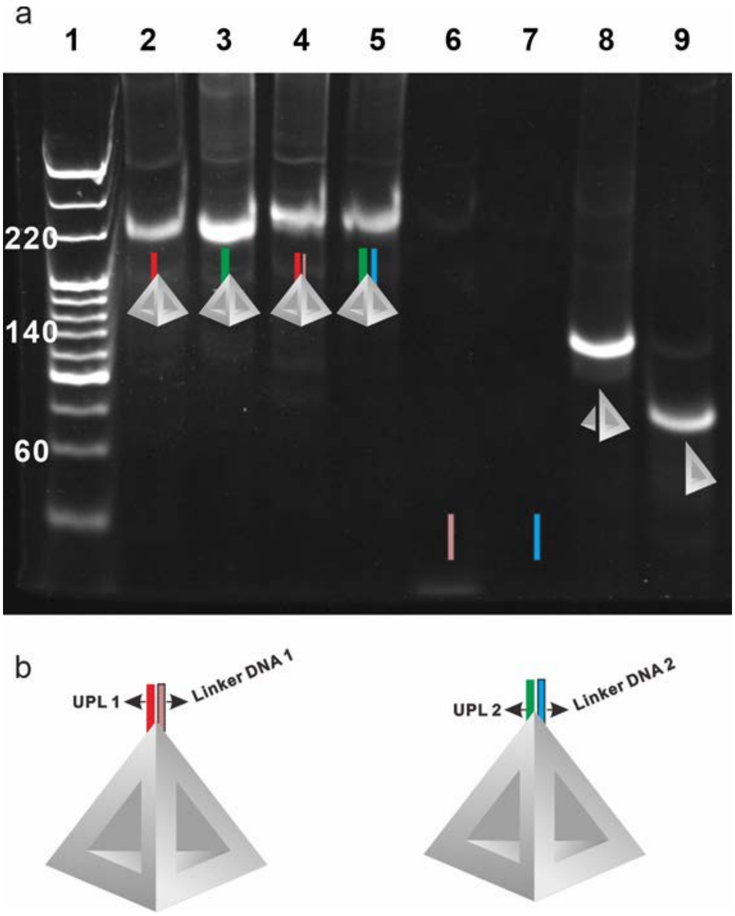

Supplementary Figure 3. DNA nanostructures assembly. (a) Gel results (PAGE electrophoresis) of DNA nanostructures. Lane 1: ladder; lane 2: A1BCD; lane 3: A2BCD; lane4: A1BCD-Linker1; lane 5: A2BCD-Linker 2; lane 6: linker 1; lane 7: linker 2; lane 8: BCD; lane 9: BC. (b) Designed nanostructure of UPL 1 and UPL 2. The source data are provided as a Source Data file.

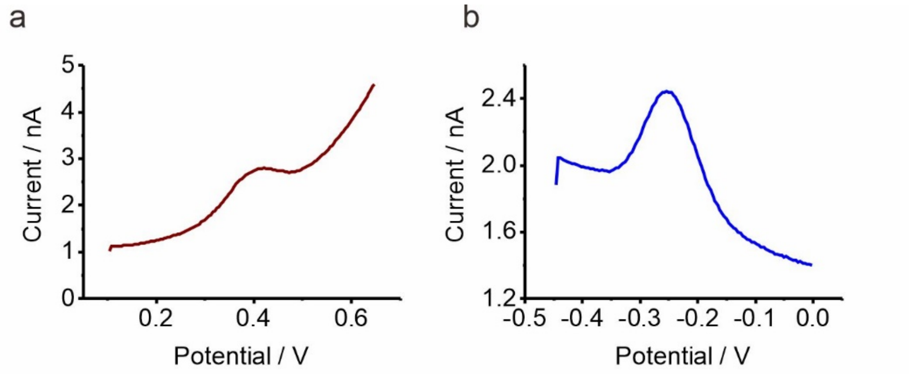

Supplementary Figure 4. Electrochemical characterization of redox labels modified DNA. The redox labels of methylene blue and ferrocene were labeled on the linker1 and linker2, respectively. When the linker-enzyme conjugates were immobilized on the TDN decorated biosensing interface, the electrochemical signals can be monitored. (a) Electrochemical signal of immobilized HRP-linker2-ferrocene. (b) Electrochemical signal of immobilized SOX-linker1-methylene blue.

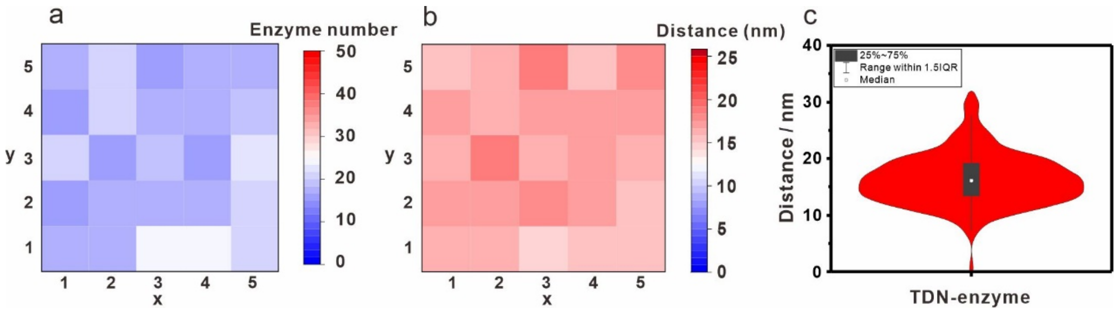

Supplementary Figure 5. TDN-enzyme distribution. We count the number of the TDN-enzyme conjugates in 25 units areas (100 nm× 100 nm for each unit area) of an AFM image of BEH (a), then derive the inter-enzyme distance from the density (b) and the statistical inter-enzyme distance distribution (c). In the boxplots the white dots represent the median value, the up and down bounds of box represent the first quartile and third quartile values and the vertical black line represent the whisker. The sample size used to derive statistics is 468 in (c). The source data are provided as a Source Data file.

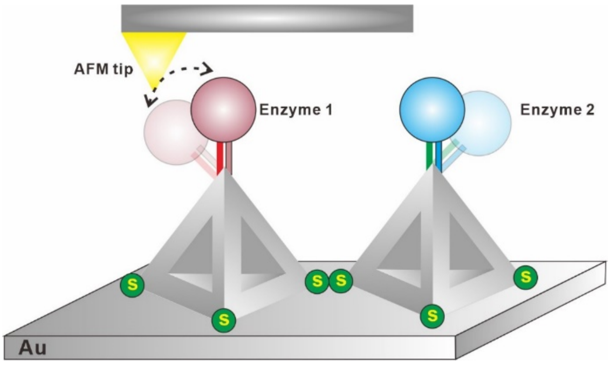

Supplementary Figure 6. AFM scanning process of TDN-enzyme. The scheme of linker flexibility effect in the AFM scanning process, which could provide larger distance between enzymes than that of the initial design.

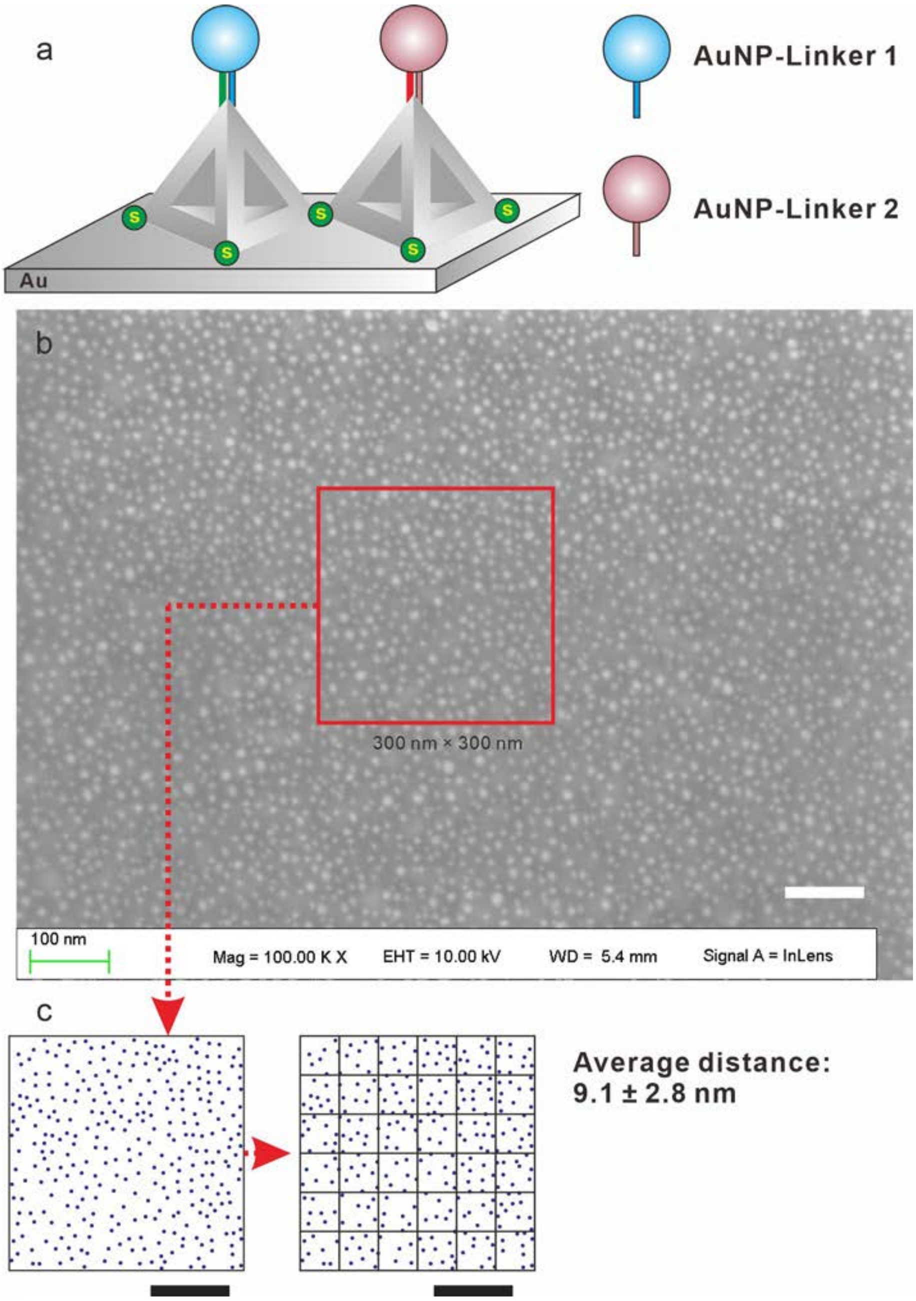

Supplementary Figure 7. TDNs-directed assembly of AuNPs (5 nm in diameter). (a) The scheme of TDNs-directed assembly structure. (b) SEM image of TDNs-directed assembly of AuNPs on gold surface. (c) We calculated the inter-particle distance of a representative region with 300 nm×300 nm, by dividing the region into a 6×6 matrix. The average distance for the whole area is 9.1±2.8 nm. Scale bar: 100 nm.

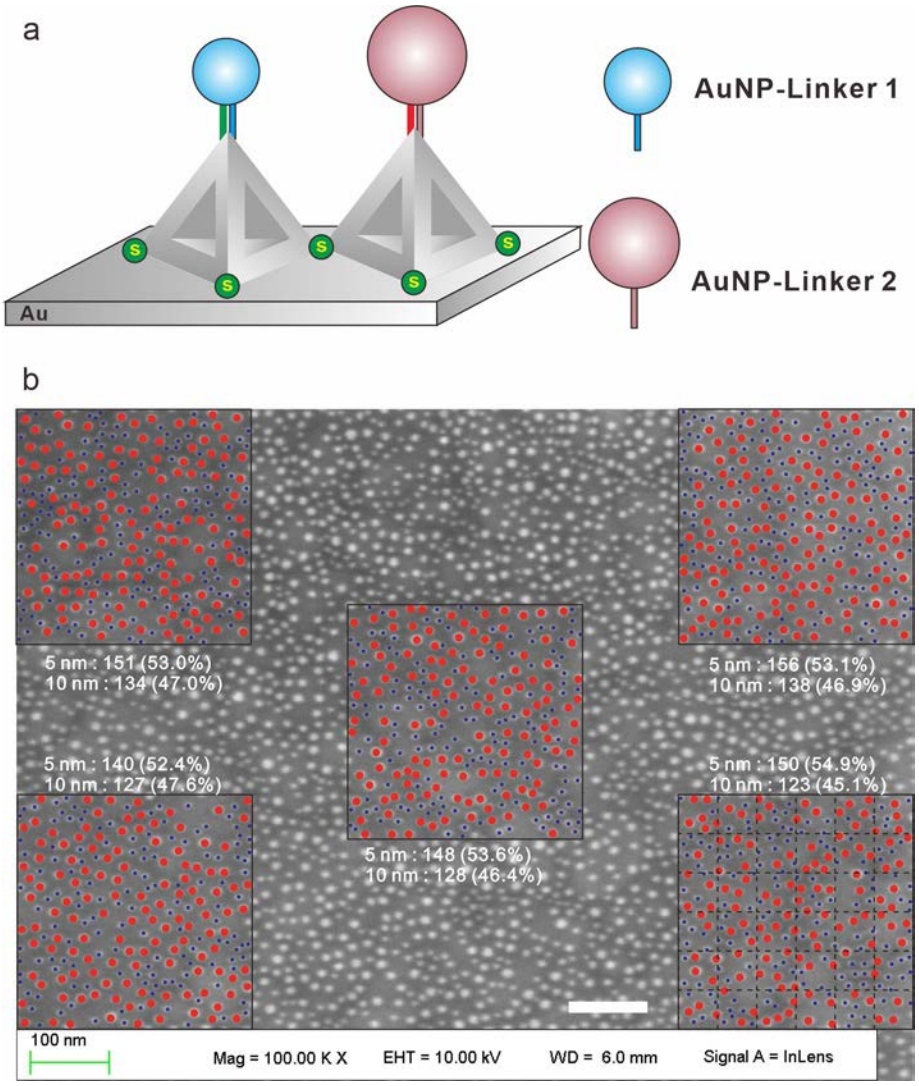

Supplementary Figure 8. TDNs-directed assembly of AuNPs (5 nm and 10 nm in diameter). (a) The scheme of TDNs-directed assembly structure. (b) SEM image of TDNs-directed assembly of AuNPs on gold surface. The number of each type of AuNP were counted in five representative area (300nm × 300 nm). The number ratios of AuNPs (5 nm) and AuNP (10 nm) were 0.89, 0.88, 0.91, 0.86, and 0.82, respectively. Red dot indicates AuNP of 10 nm; Blue dot indicates AuNP of 5 nm. Scale bar: 100 nm.

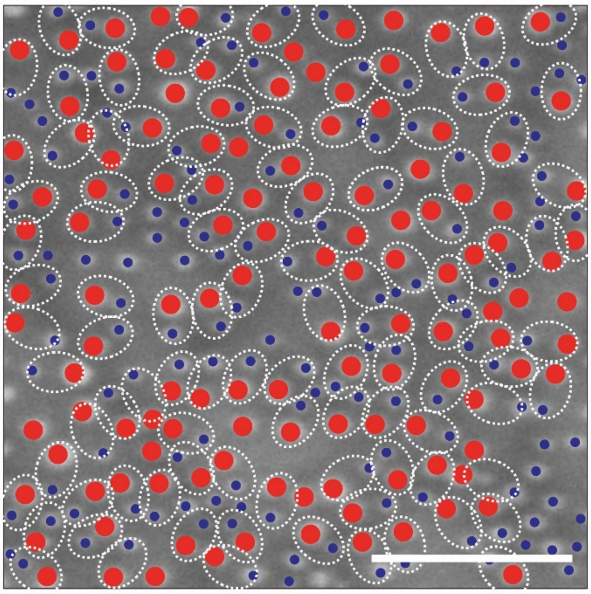

Supplementary Figure 9. Investigation of the AuNP (5 nm)-AuNP (10 nm) hetero-pairs in the SEM image (300nm × 300 nm). We observed minor discrepancy between 5-nm AuNPs (blue dot) and 10-nm AuNPs (red dot), which possibly arises from the incomplete formation of the AuNP (5 nm)-AuNP (10 nm) pairs. The yield of the heterogeneous pairs was estimated to be ~88%. Red dot indicates AuNP of 10 nm; Blue dot indicates AuNP of 5 nm. Scale bar: 100 nm. The sample size used to derive statistics is 276.

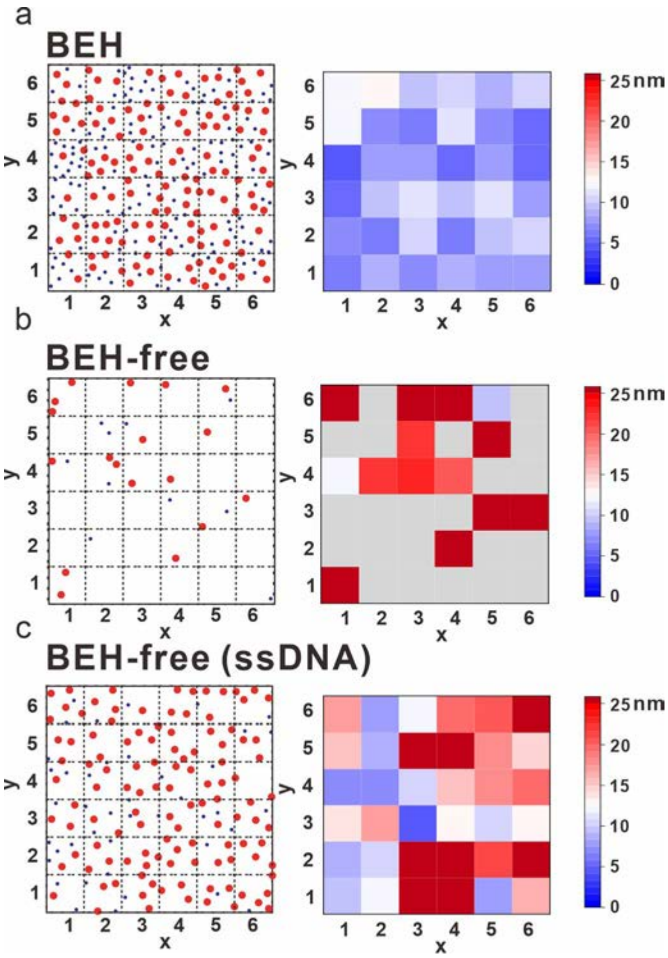

Supplementary Figure 10. The inter-particle distance of AuNP -AuNP hetero-pairs. (a) The inter-particle distance obtained by the BEH method with TDN of 17 bp was ~ 8.5 ± 3.8nm. The inter-particle distance obtained by (b) naked gold surface (~ 49.7 ± 25.6 nm) and (c) ssDNA (~ 19.3 ± 12.2 nm) modified gold surface were significantly larger than 10 nm. Red dot indicates AuNP of 10 nm; Blue dot indicates AuNP of 5 nm. The sample size used to derive statistics is 285, 29 and 157 in (a), (b) and (c).

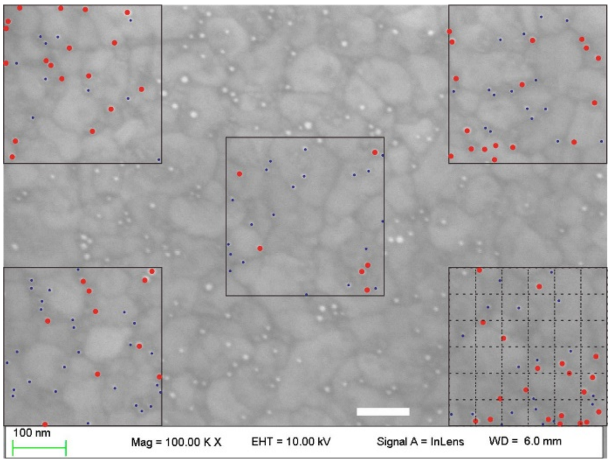

Supplementary Figure 11. The SEM image of AuNPs on naked gold surface. AuNPs with different diameter (5 nm and 10 nm) were assembled on naked gold surface, which is BEH-free method. Red dot indicates AuNP of 10 nm; Blue dot indicates AuNP of 5 nm. Scale bar: 100 nm.

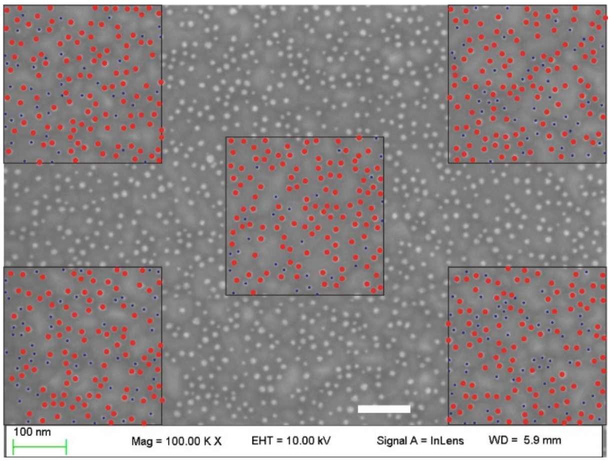

Supplementary Figure 12. The SEM image of AuNPs on ssDNA modified gold surface. AuNPs with different diameter (5 nm and 10 nm) were assembled on ssDNA modified gold surface, which is BEH-free (ssDNA) method. Red dot indicates AuNP of 10 nm; Blue dot indicates AuNP of 5 nm. Scale bar: 100 nm.

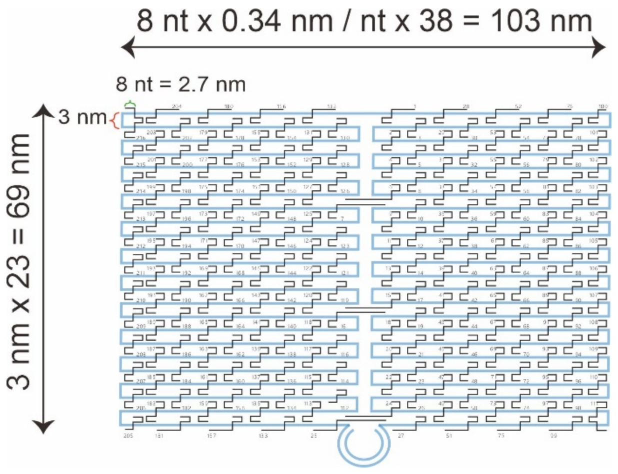

Supplementary Figure 13. The design of origami and information of positions of staple strands.

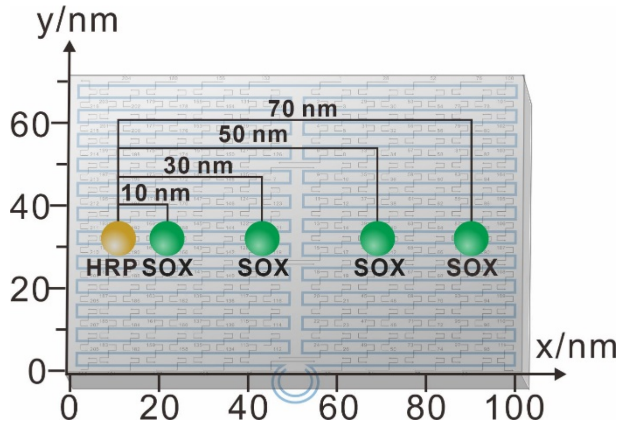

Supplementary Figure 14. The design of origami and the corresponding inter-enzyme distances between HRP and SOX.

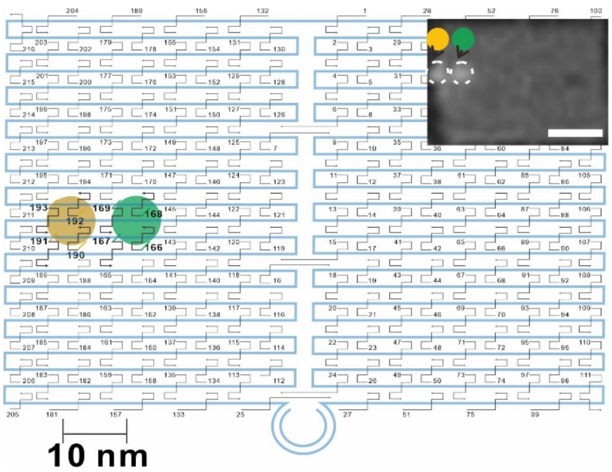

Supplementary Figure 15. The design of G1 with inter-enzyme distance of 10 nm. For HRP anchoring on G1, we fixed the HRP (yellow dot) in position 1 with strands (190, 191, 192, 193). For SOX anchoring on G1 (green dot), our designed staple DNAs were new-169, new-167, new-168 and new-166 instead of normal 169, 167, 168 and 166. The inset picture is the AFM image of DNA origami with the anchored HRP and SOX. Scale bar: 30 nm.

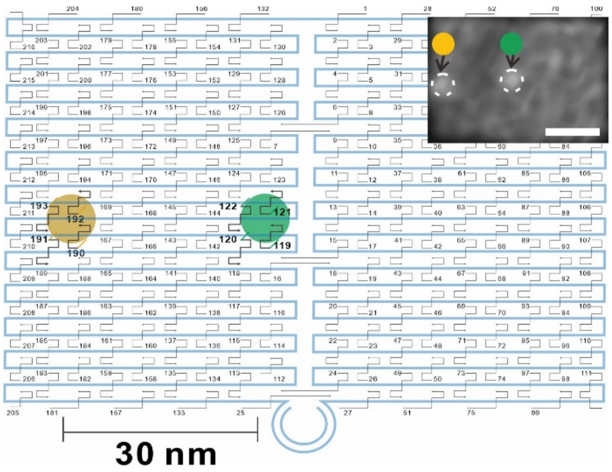

Supplementary Figure 16. The design of G3 with inter-enzyme distance of 30 nm. For HRP anchoring on G3, we fixed the HRP (yellow dot) in position 1 with strands (190, 191, 192, 193). For SOX anchoring on G3 (green dot), our designed staple DNAs were new-122, new-120, new-121 and new-119 instead of 122, 120, 121 and 119. The inset picture is the AFM image of DNA origami with the anchored HRP and SOX. Scale bar: 30 nm.

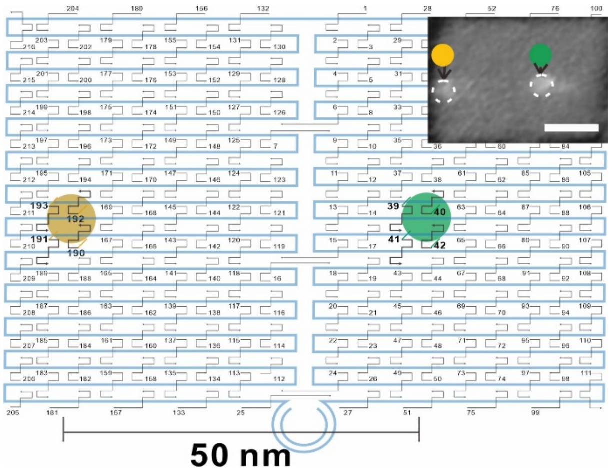

Supplementary Figure 17. The design of G5 with inter-enzyme distance of 50 nm. For HRP anchoring on G5, we fixed the HRP (yellow dot) in position 1 with strands (190, 191, 192, 193). For SOX anchoring on G5 (green dot), our designed staple DNAs were new-39, new-40, new-41 and new-42 instead of 39, 40, 41 and 42. The inset picture is the AFM image of DNA origami with the anchored HRP and SOX. Scale bar: 30 nm.

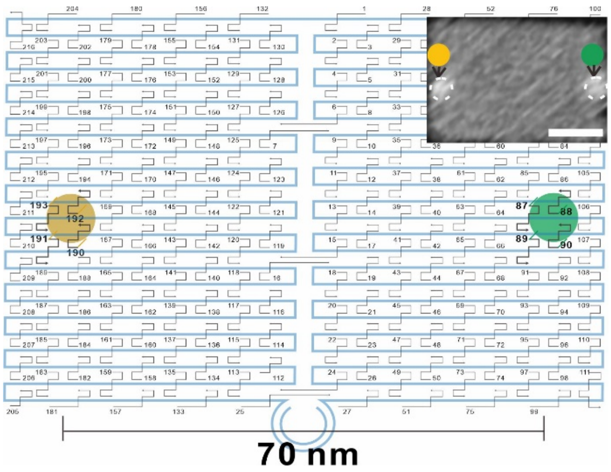

Supplementary Figure 18. The design of G7 with inter-enzyme distance of 70 nm. For HRP anchoring on G7, we fixed the HRP (yellow dot) in position 1 with strands (190, 191, 192, 193). For SOX anchoring on G7 (green dot), our designed staple DNAs were new 87, new 89, new 88 and new 90 instead of 87, 89, 88 and 90. The inset picture is the AFM image of DNA origami with the anchored HRP and SOX. Scale bar: 30 nm.

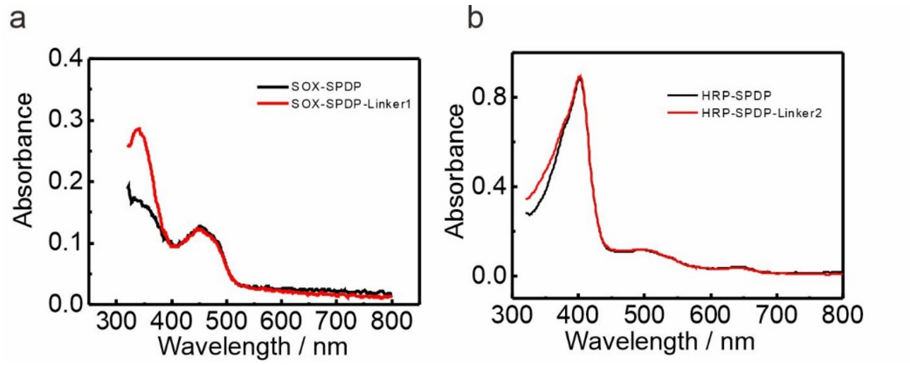

Supplementary Figure 19. Quantification of protein-DNA conjugation efficiency via absorbance spectra. (a) SOX-linker1 conjugation: ΔA 343 before and after linker1 conjugation is ~ 0.112, corresponding to 15 µM linker1 coupled with 8.5 µM SOX (ε=12700 M^{-1} cm^{-1} at 450 nm for SOX). (b) Linker2 conjugation: ΔA 343 before and after linker2 conjugation is ~ 0.088 (extinction coefficient: 8080 M^{-1} cm^{-1}), corresponding to 11.4 µM linker2 coupled with 8.92 µM HRP (ε=100000 M^{-1} cm^{-1} at 403 nm for HRP).

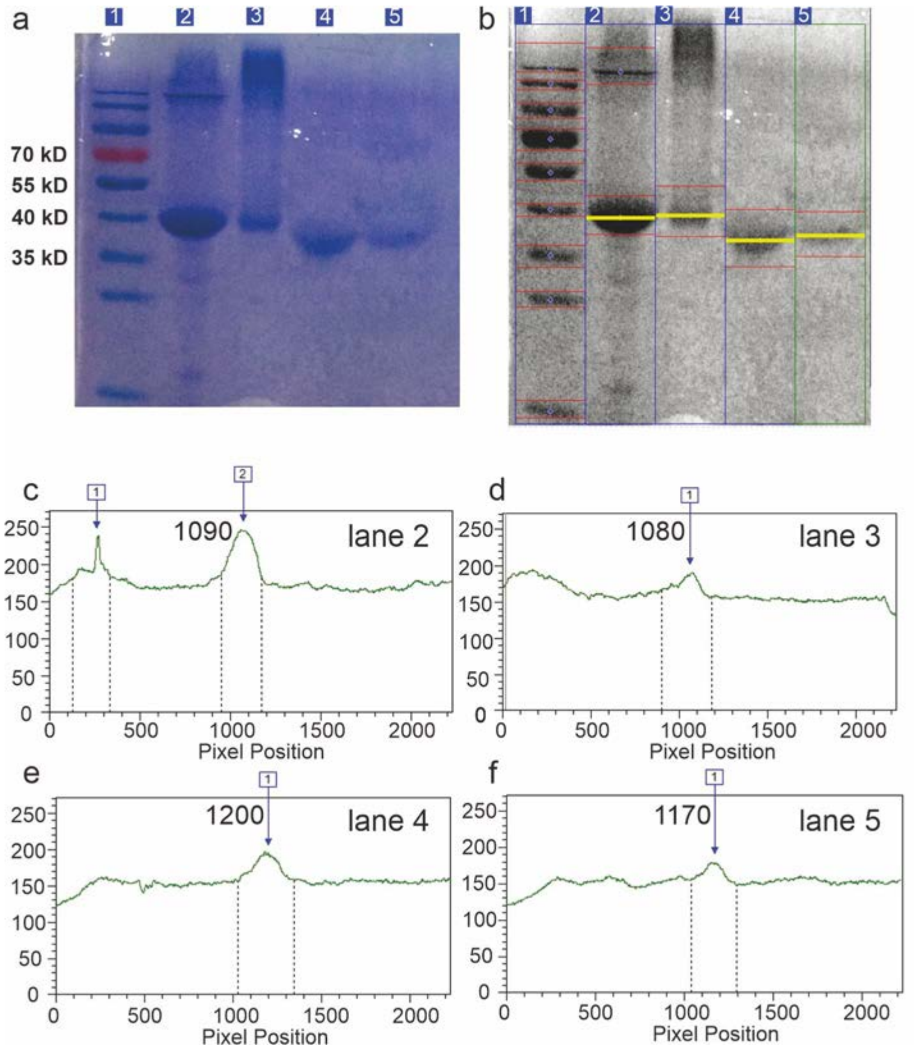

Supplementary Figure 20. SDS-PAGE electrophoresis of enzyme-DNA conjugates. (a) lane1: ladder; lane 2: SOX; lane 3: SOX-linker1; lane 4: HRP; lane5: HRP-linker2. Conditions: NuPAGE 5%-12% Bis Tris Gel with a constant voltage of 150 V for 70 mins. (b) gray scale figure of a), all the squares are analyzed from ImageQuant TL 1D v8.1 software to give the peak of the band. (c) to (f) are the peak detail information of different lanes from (b). x axis is the pixel position which is consistent with different shift position. Lane 3 of SOX-linker1 is a little bit delay of 1080 compared with lane2 of SOX with 1090. In addition, HRP-linker2 with 1170 compared with HRP with 1200, so this shows both SOX and HRP are successfully linked with DNA linker1 and linker2, respectively. The source data are provided as a Source Data file.

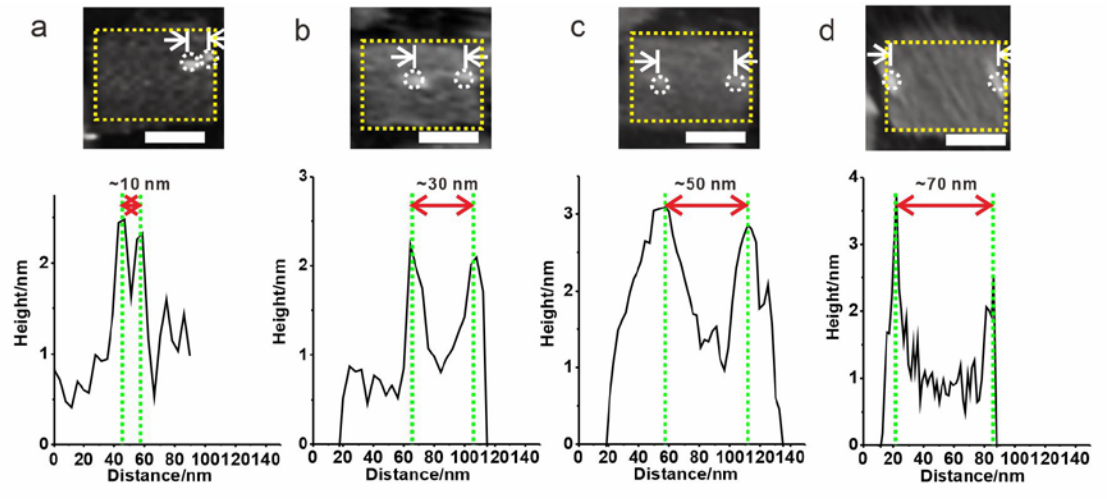

Supplementary Figure 21. AFM images and measurements of enzyme distance. The precisely controlled distance between two enzymes on origami including (a) 10 nm, (b) 30 nm, (c) 50 nm and (d) 70 nm. Dotted rectangles represent the edge of designed DNA origami. Scale bar: 50 nm.

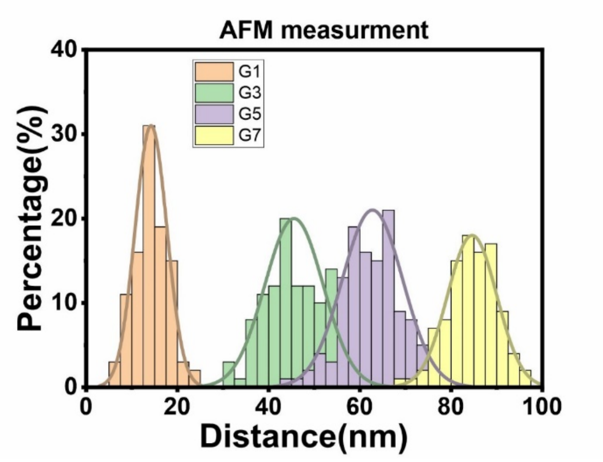

Supplementary Figure 22. Statistical distribution of inter-enzyme distance obtained from AFM images. The sample size used to derive statistics is 432. The source data are provided as a Source Data file.

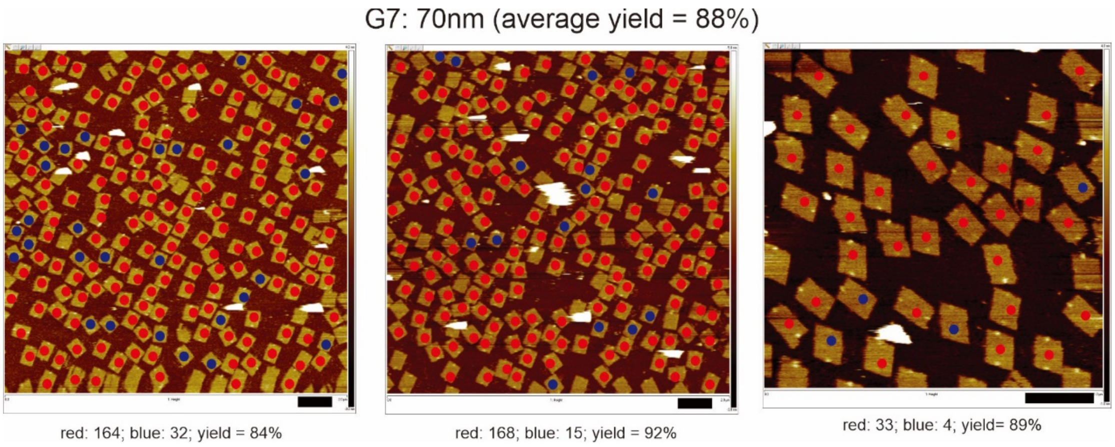

Supplementary Figure 23. The AFM images of G7 in large scale and the calculation of the yield of G7. The average yield of G7 is ~88%. (Red dot indicates two enzymes co-assembled on one origami with expected distance, and blue dot indicates DNA origamis missing one enzyme or two enzymes). Scale bar: 200 nm. The sample size used to derive statistics is 416.

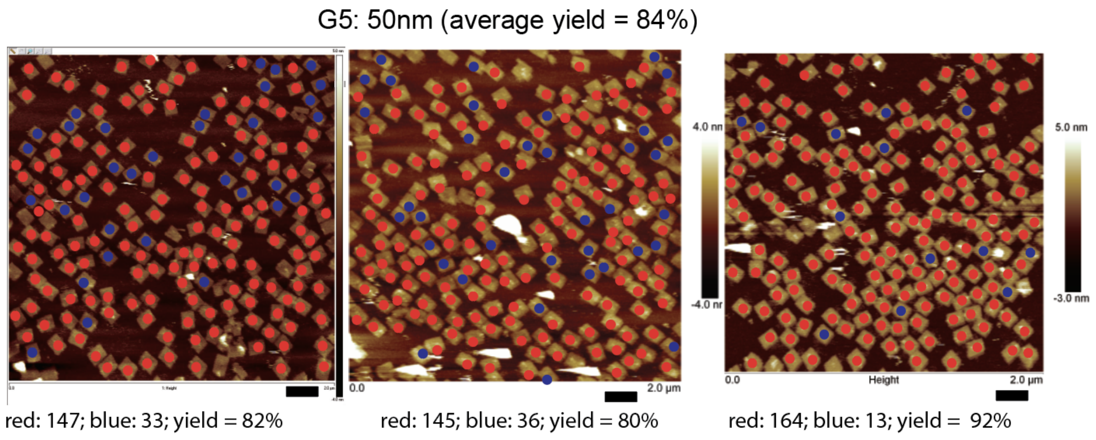

Supplementary Figure 24. The AFM images of G5 in large scale and the calculation of the yield of G5. The average yield of G5 is ~84%. (Red dot indicates two enzymes co-assembled on one origami with expected distance, and blue dot indicates DNA origamis missing one enzyme or two enzymes). Scale bar: 200 nm. The sample size used to derive statistics is 538.

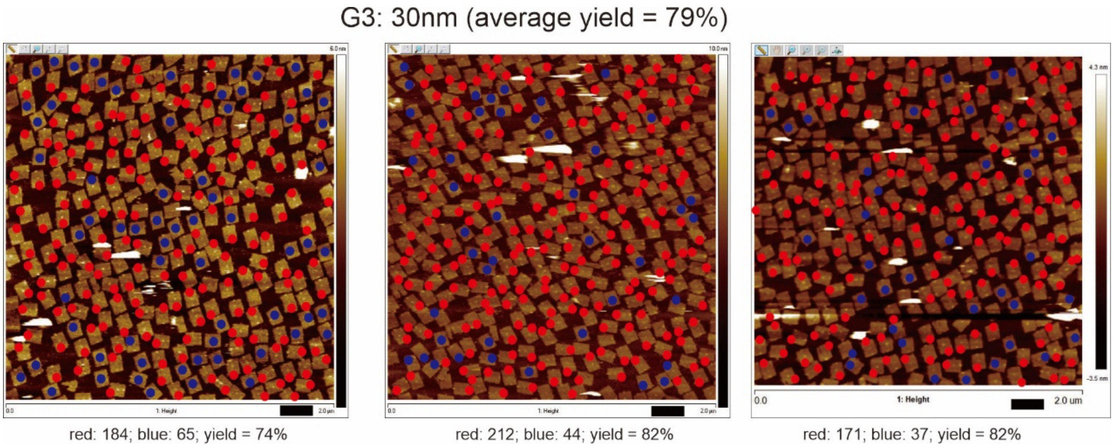

Supplementary Figure 25. The AFM images of G3 in large scale and the calculation of the yield of G3. The average yield of G3 is ~79%. (Red dot indicates two enzymes co-assembled on one origami with expected distance, and blue dot indicates DNA origamis missing one enzyme or two enzymes). Scale bar: 200 nm. The sample size used to derive statistics is 713.

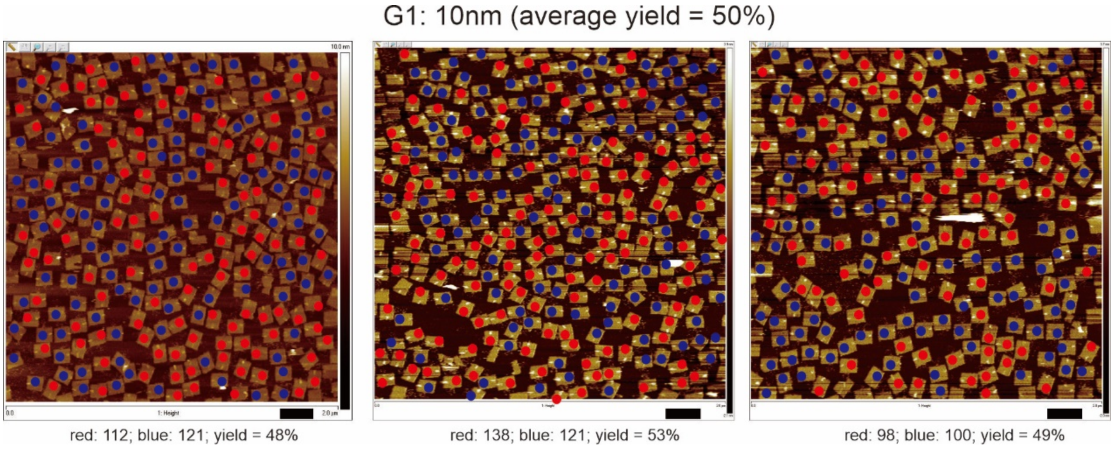

Supplementary Figure 26. The AFM images of G1 in large scale and the calculation of the yield of G1. The average yield of G1 is ~50%. (Red dot indicates two enzymes co-assembled on one origami with expected distance, and blue dot indicates DNA origamis missing one enzyme or two enzymes). Scale bar: 200 nm. The sample size used to derive statistics is 690.

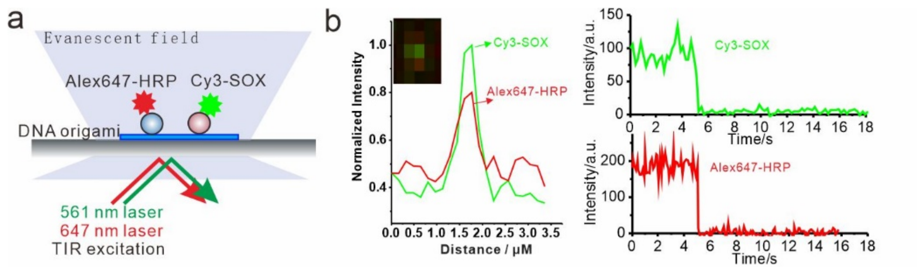

Supplementary Figure 27. Co-localization studies of both SOX and HRP on a single rectangular origami. (a) Scheme of co-localization methods for Alex647 linked HRP and Cy3 linked SOX on DNA origami. (b) Colocalized fluorescence spectrum of HRP and SOX on DNA origami, which formed the basis of enzyme heterojunction and cascade reaction.

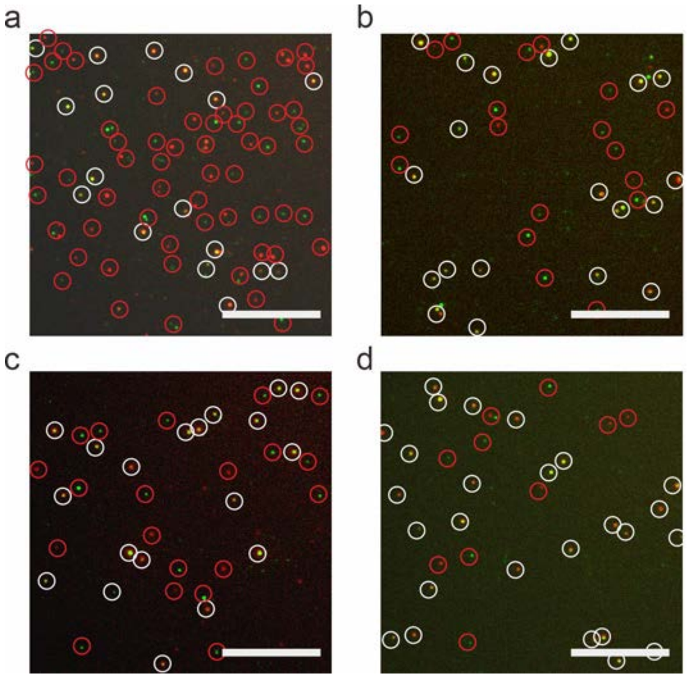

Supplementary Figure 28. Co-localization studies of anchoring of both SOX and HRP on a single rectangular origami. (a-d) Co-localization results for G1 (10 nm), G3 (30 nm), G5 (50 nm) and G7 (70 nm), respectively. SOX was modified with Cy3 and HRP was modified with Alex-647. Scale bar: 10 μm.

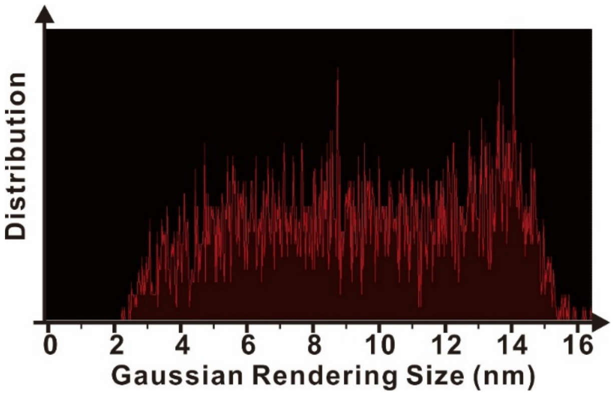

Supplementary Figure 29. The gaussian rendering size acquired in Nikon N-STROM system after signal peak height filtering and drift correction.

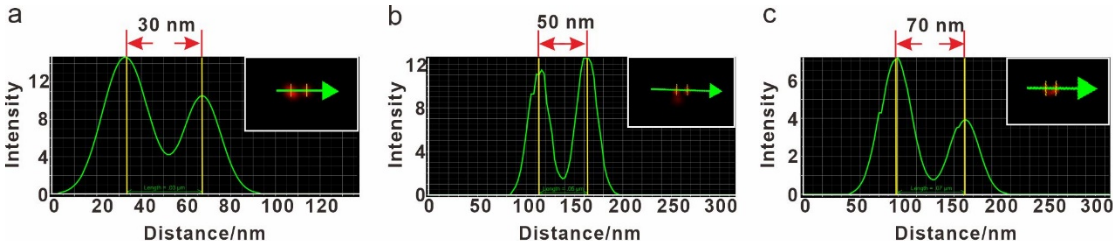

Supplementary Figure 30. STORM super-resolution analysis of enzymes distance. We measured the inter-enzyme distance between SOX and HRP of (a) 30 nm, (b) 50 nm and (c) 70 nm, and obtained consistent inter-enzyme distance with our designed distance. The green arrows represent the measurement direction in each SOX/HRP pair. The green peaks represent the positions of fluorescence dots (SOX or HRP) and the inter-enzyme distance was measured with yellow vertical lines.

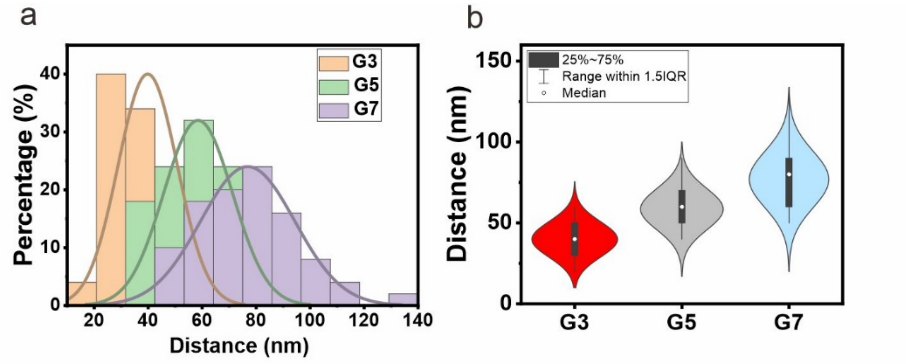

Supplementary Figure 31. Statistical distribution of inter-enzyme distance obtained from co-localization images by STORM method. The distances distribution of G3, G5 and G7 groups are shown as (a) histogram and (b) violin plot. In the boxplots the white dots represent the median value, the up and down bounds of box represent the first quartile and third quartile values and the vertical black line represent the whisker. The sample size used to derive statistics is 56, 54 and 52. The source data are provided as a Source Data file.

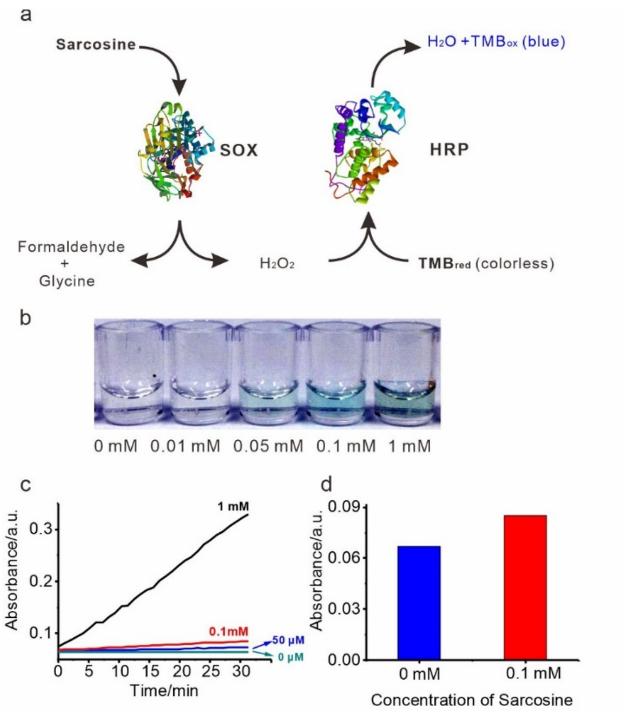

Supplementary Figure 32. Design and evaluation of the enzyme cascade for sarcosine detection in solution phase. In our design, SOX catalyzes the oxidative demethylation of sarcosine to yield H_2O_2, which is sequentially catalyzed by horse radish peroxidase (HRP). This enzyme cascade can be visualized by the peroxidase-coupled color reaction with a chromogenic substrate-TMB (3,3',5,5'-Tetramethylbenzidine, from colorless to blue), which can be monitored by UV-vis spectrum. We observed the remarkable color change by adding sarcosine (1 mM) to initiate the cascade reaction, which testified the feasibility of our design. The color change degraded with the decrease of sarcosine concentration, indicating the enzyme cascade reaction is sarcosine dependent. To quantify the enzyme cascade reaction, we monitored the adsorption at 652 nm. We can quantitatively detect sarcosine from 50 μM to 1 mM by unaided eyes or by UV-vis spectroscopy. (a) The design of enzyme cascade for sarcosine detection. In our design, SOX was selected to catalyze the oxidative demethylation of sarcosine, yielding the H_2O_2. HRP was then employed to catalyze the H_2O_2 to H_2O. With the color change of co-substrate TMB, the enzyme cascade reaction can be monitored by unaided eyes or UV-vis spectroscopy. (b) The color of the solution changed from colorless to blue with various concentrations of sarcosine, which can be quantitatively monitored by the UV-vis adsorption at 652 nm (c). (d) The comparison of adsorption of enzyme cascade reaction with sarcosine (0.1 mM) and without sarcosine. The source data are provided as a Source Data file.

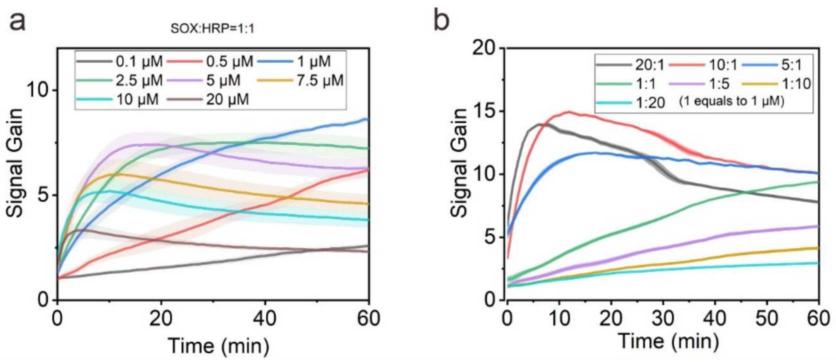

Supplementary Figure 33. Optimization of the enzyme cascade reaction in solution. (a) The selection of the optimized enzyme concentration under the SOX:HRP=1:1. (b) The selection of the optimized enzyme ratio, indicating the optimized ratio of 10:1 (SOX:HRP). Error bars represent the mean ± s.d. of triplicates. The source data are provided as a Source Data file.

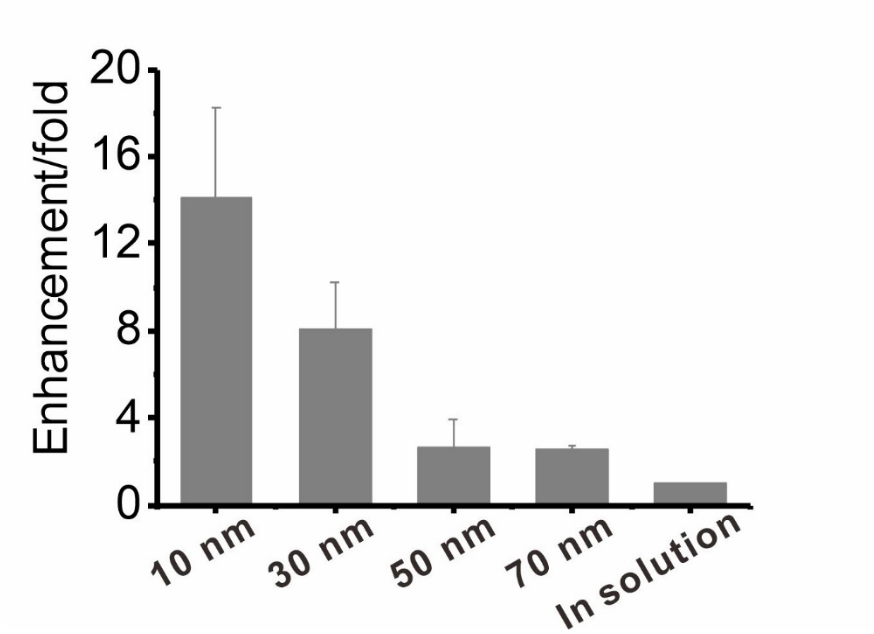

Supplementary Figure 34. Enhancement of the activity of enzyme pairs on DNA origami compared to the free enzyme in solution. Error bars represent the mean ± s.d. of quintuplicates. The source data are provided as a Source Data file.

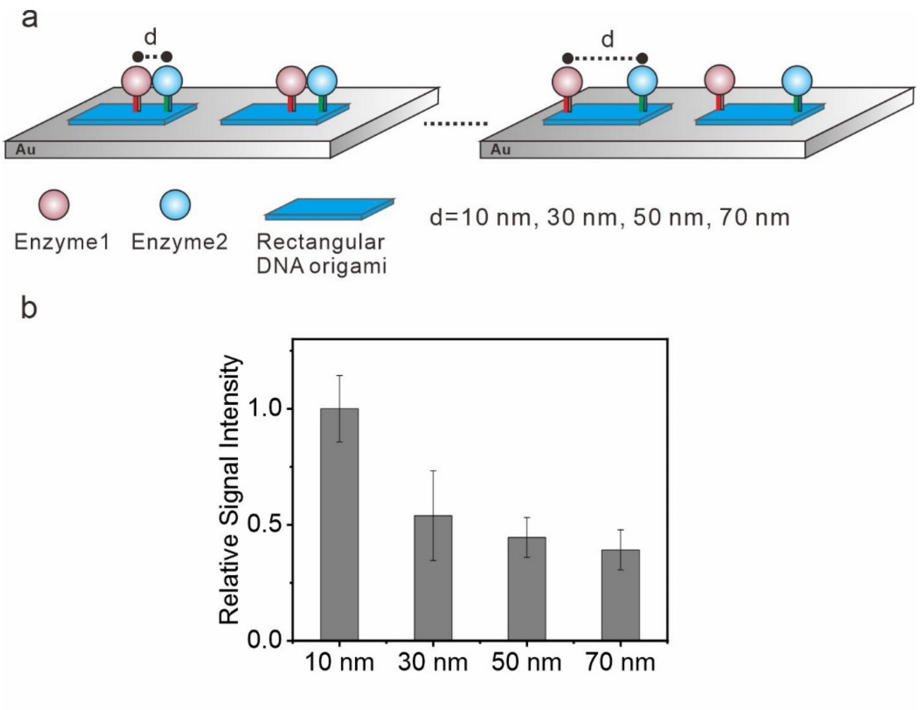

Supplementary Figure 35. Inter-enzyme distance-dependent cascade efficiency on the electrode. (a) The enzyme pair for enzyme cascade (enzyme 1 and enzyme 2) were site-specifically conjugated on rectangular DNA origami, which were then immobilized on the gold electrode surface via the thiol groups linked on the DNA origami. (b) The relative signal intensity decreased along with the increase of inter-enzyme distance, indicating the inter-enzyme distance dependent enzyme cascade efficiency on the gold electrode surface. Error bars represent the mean ± s.d. of triplicates. The source data are provided as a Source Data file.

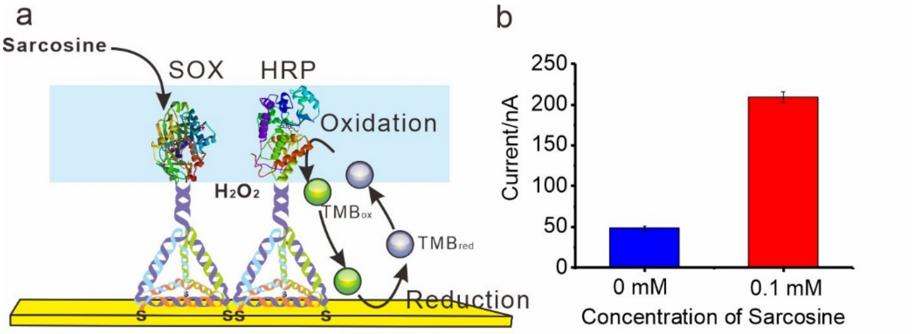

Supplementary Figure 36. The TDNs-enzyme immobilized on the gold surface. (a) The SOX and HRP were attached on the TDNs by the hybridization of linker DNA and UPL. The distance between enzymes was well controlled by the TDNs to catalyze the in-situ generated intermediate H_2O_2. (b) The signal increase in the presence of sarcosine (0.1 mM) was improved to 335%. Error bars represent the mean ± s.d. of triplicates. The source data are provided as a Source Data file.

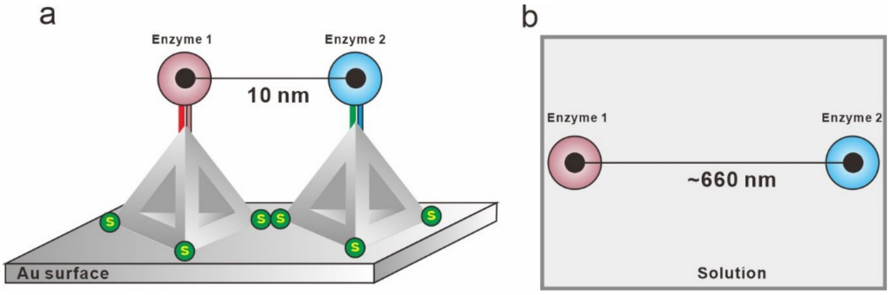

Supplementary Figure 37. Scheme of enzymes distance. Comparison of inter-enzyme distance on BEH electrode (a) and in solution (b).

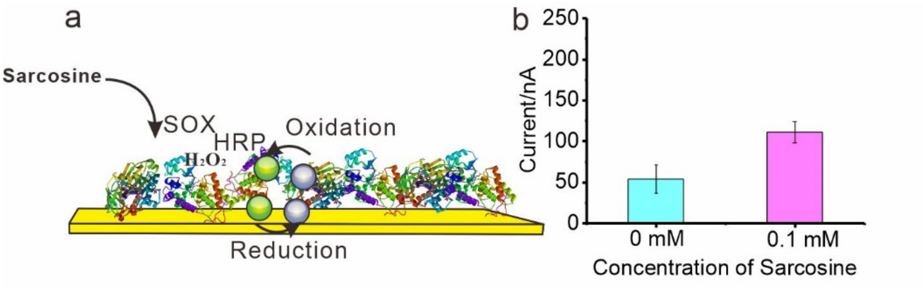

Supplementary Figure 38. The enzymes adsorbed on the gold surface. (a) The enzymes were immobilized on the electrode surface using BEH-free method by adsorption. (b) The sensor performance was suppressed with a relatively lower signal to background ratio at 0.1 mM of sarcosine. Error bars represent the mean ± s.d. of triplicates. The source data are provided as a Source Data file.

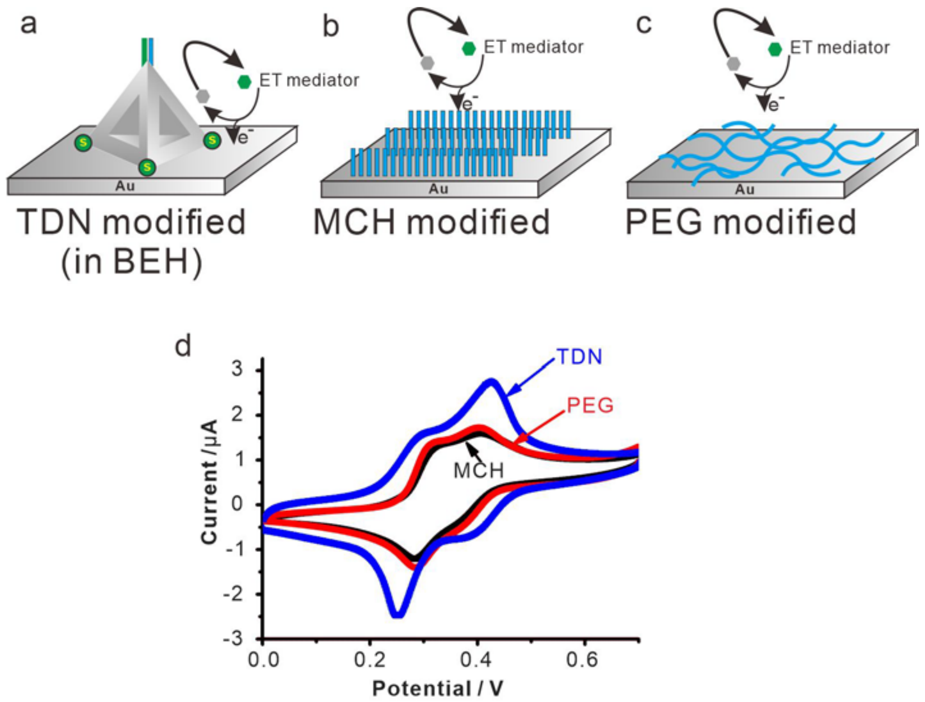

Supplementary Figure 39. Electronic transport on electrode surface. The scheme of (a) TDN, (b) 6-Mercapto-1-hexanol (MCH) and (c) Polyethylene Glycol (PEG) modified electrode and the (d) cyclic voltammetry graphs of redox mediator (TMB) on these modified electrodes.

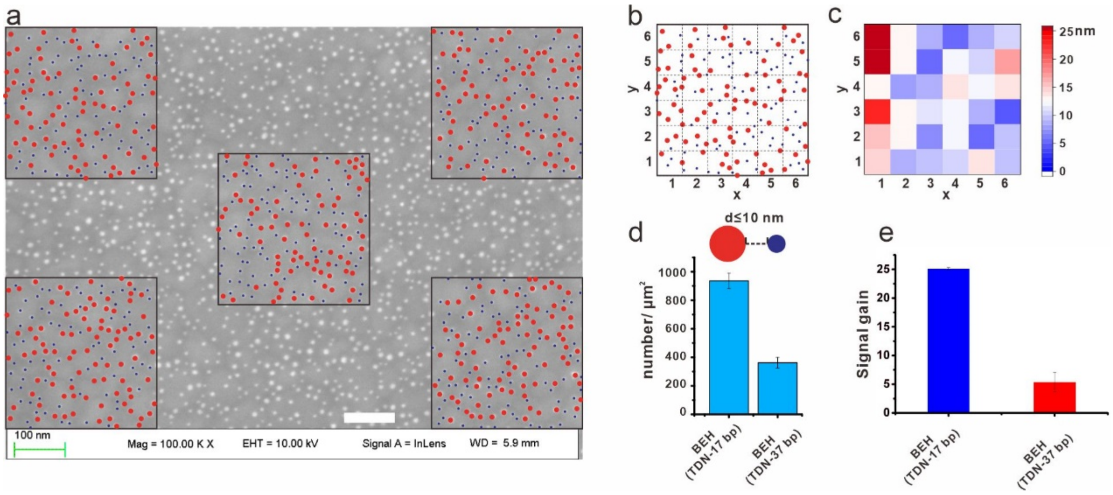

Supplementary Figure 40. AuNPs assembly using TDNs of 37 bp. (a) SEM image of the assembly of AuNPs (5 nm and 10 nm in diameter) on gold surface by using BEH with TDNs of 37 bp. (b and c) The inter-particle distance of AuNP (5 nm)-AuNP (10 nm) pairs was measured. The inter-particle distance obtained by the BEH method with TDN of 37 bp was 12.8±7.3 nm. (d) The number of AuNP (5 nm)-AuNP (10 nm) pairs with inter-particle distance < 10 nm were significantly lower than that of BEH with TDN of 17 bp. (e) The signal gain to detect sarcosine of 100 mM using BEH with TDN of 17 bp and 37 bp, respectively. Red dot indicates AuNP of 10 nm; Blue dot indicates AuNP of 5 nm. Error bars represent the mean ± s.d. of quintuplicates for (d) and triplicates for (e). The source data are provided as a Source Data file.

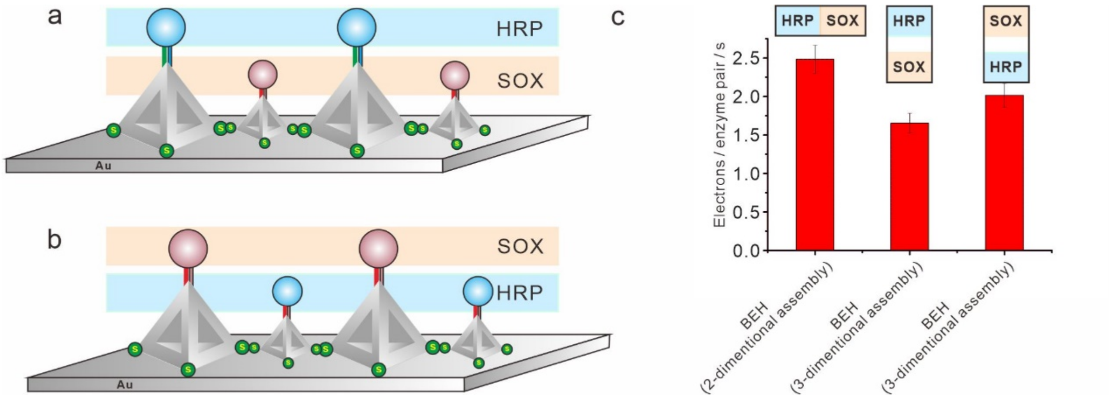

Supplementary Figure 41. Electrons transport difference on different structured electrode surface. The comparison of signal increase of sarcosine detection using 2-dimensional assembly and 3-dimentional assembly (HRP monolayer with a layer of the sarcosine oxidase on top, and sarcosine oxidase monolayer with a HRP layer on top). (a) Scheme of assembly of HRP monolayer with a layer of the sarcosine oxidase on top. (b) Scheme of assembly of HRP monolayer with a layer of the sarcosine oxidase on top. (c) Electron transfer of one SOX/HRP pair in 1 second was obtained using BEH (2-dimensional assembly) and BEH (3-dimensional assembly, HRP monolayer with a layer of the sarcosine oxidase on top, and sarcosine oxidase monolayer with a HRP layer on top) to detect sarcosine of 10 mM. Error bars represent the mean ± s.d. of triplicates. The source data are provided as a Source Data file.

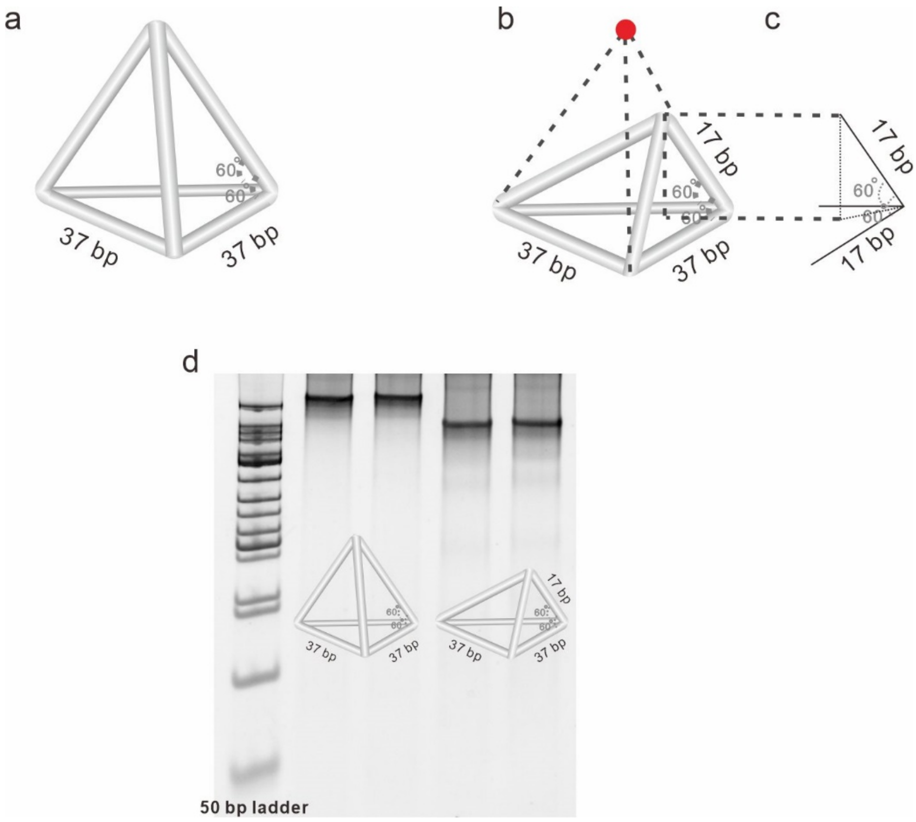

Supplementary Figure 42. The scheme and synthesis of the TDN. (a) a conventional design of a TDN (37 bp). (b) The design of TDN (37-17), which has the equal height with TDN (17 bp) and larger edge length (37 bp in edge length). (c) The height of TDN (17 bp), which is equal to TDN (37-17). (d)The gel analysis of the successful synthesis of TDN (37-17). Lane 1: DNA ladder; Lane 2: TDN (37 bp)-UPL1; Lane 3: TDN (37 bp)-UPL2; Lane 4: TDN (37-17)-UPL1; Lane 5: TDN (37-17)-UPL2.

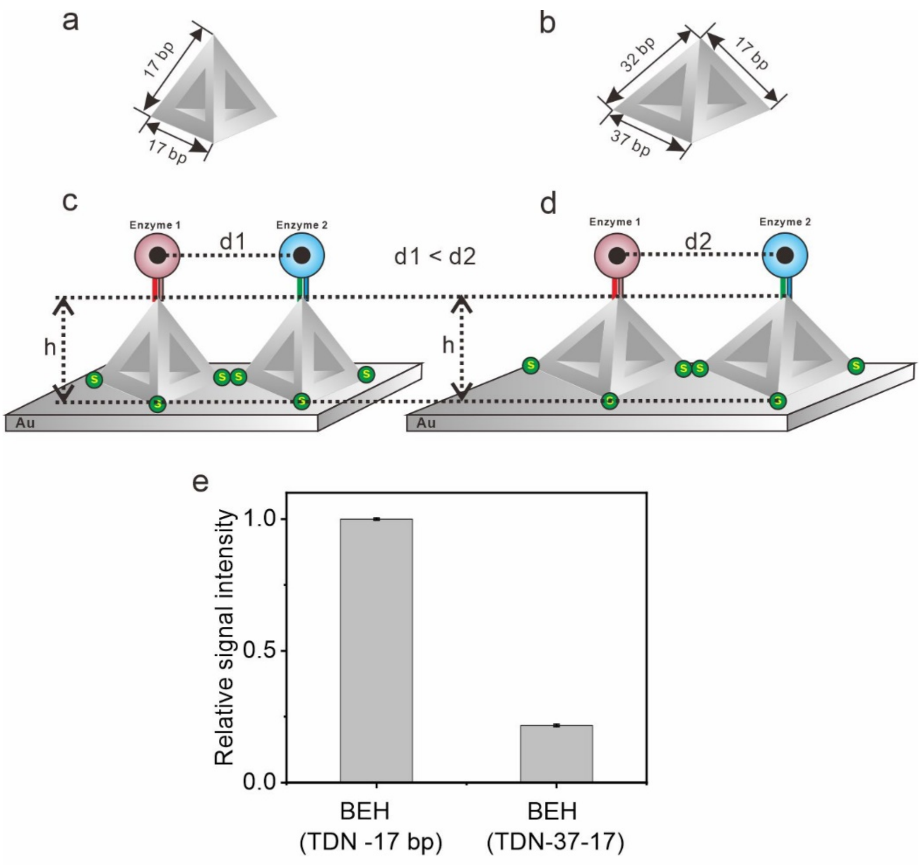

Supplementary Figure 43. The comparison of BEH catalytic signal using TDN (17 bp) and TDN (37-17). (a) The scheme of TDN (17 bp). (b) The scheme of TDN (37-17). (c and d) The scheme of BEH using TDN (17 bp) and TDN (37-17). The height between enzyme and gold electrode is equal in these two systems. (e) The relative signal obtained from the BEH using TDN (17 bp) is higher than that of the BEH using TDN (37-17). Error bars represent the mean ± s.d. of triplicates. The source data are provided as a Source Data file.

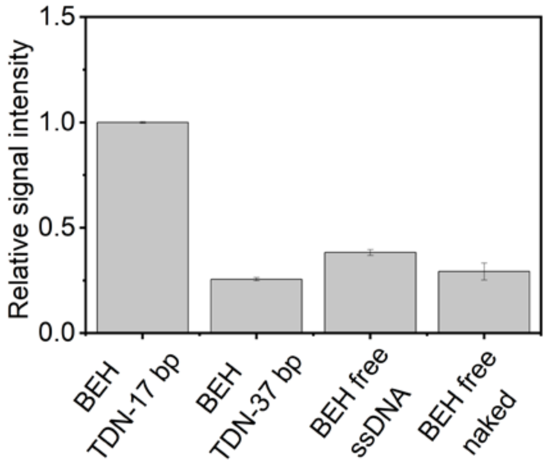

Supplementary Figure 44. The comparison of enzyme cascade catalytic signal on gold electrode. Enzymes were immobilized on TDN (17 bp), TDN (37 bp), ssDNA and naked gold electrode. Error bars represent the mean ± s.d. of triplicates. The source data are provided as a Source Data file.

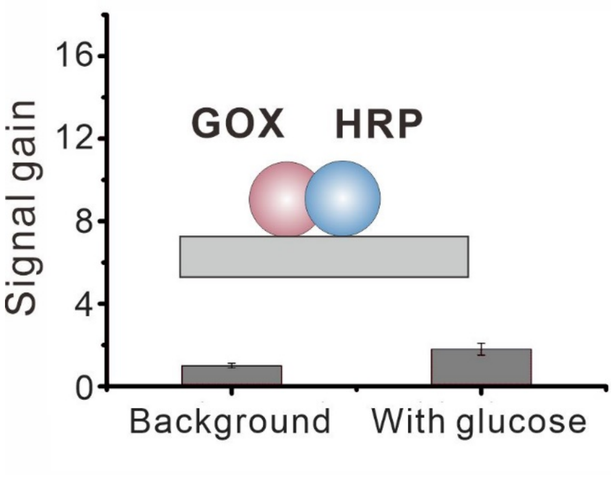

Supplementary Figure 45. Cascade reaction signal gain of traditional BEH-free method. The signal gain of TDN programmed BEH was ~ 14.7-fold in the presence of 1mM of glucose. This was in sharp contrast to the 1.7-fold of traditional BEH-free method. Error bars represent the mean ± s.d. of triplicates. The source data are provided as a Source Data file.

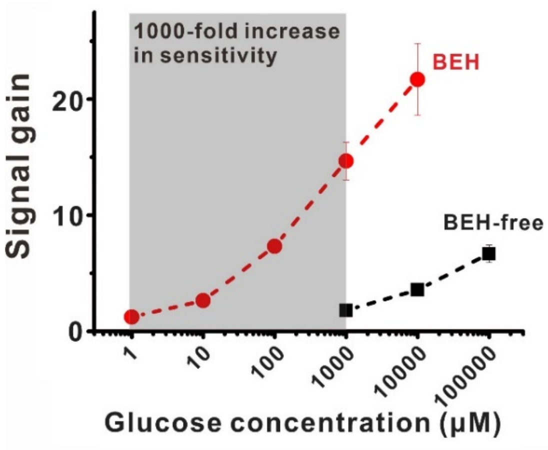

Supplementary Figure 46. Comparison of the sensitivity of glucose detection by using BEH method and BEH-free method. The GOX-HRP cascade system was used in the detection. The sensitivity of BEH to sense glucose is 1000-fold more sensitive than the BEH-free enzyme interface. Error bars represent the mean ± s.d. of triplicates. The source data are provided as a Source Data file.

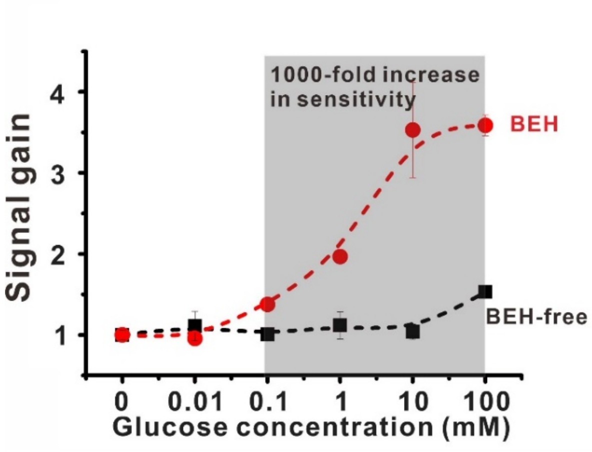

Supplementary Figure 47. Comparison of the sensitivity of glucose detection by using BEH method and BEH-free method. The GOX-G quardruplex cascade system was used in the detection. Error bars represent the mean ± s.d. of triplicates. The source data are provided as a Source Data file.

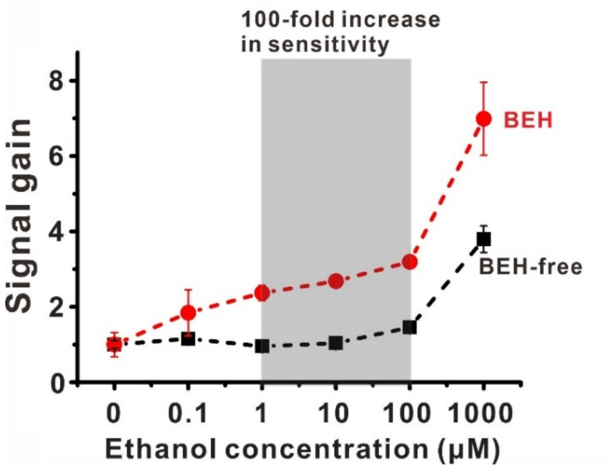

Supplementary Figure 48. Comparison of the sensitivity of ethanol detection by using BEH method and BEH-free method. The AOX-HRP cascade system was used in the detection. Error bars represent the mean ± s.d. of triplicates. The source data are provided as a Source Data file.

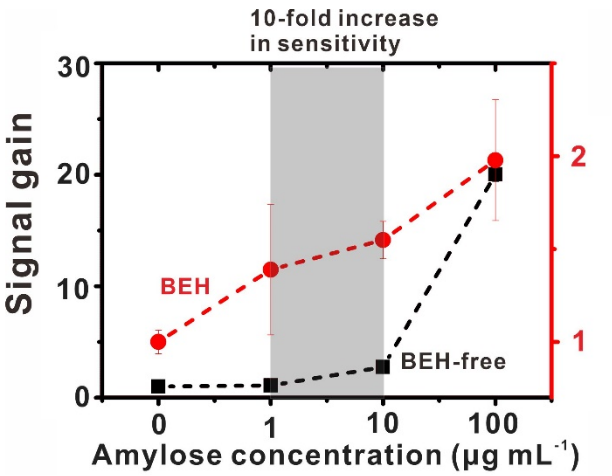

Supplementary Figure 49. Comparison of the sensitivity of amylose detection by using BEH method and BEH-free method. The AM-AOX-HRP cascade system was used in the detection. Error bars represent the mean ± s.d. of triplicates. The source data are provided as a Source Data file.

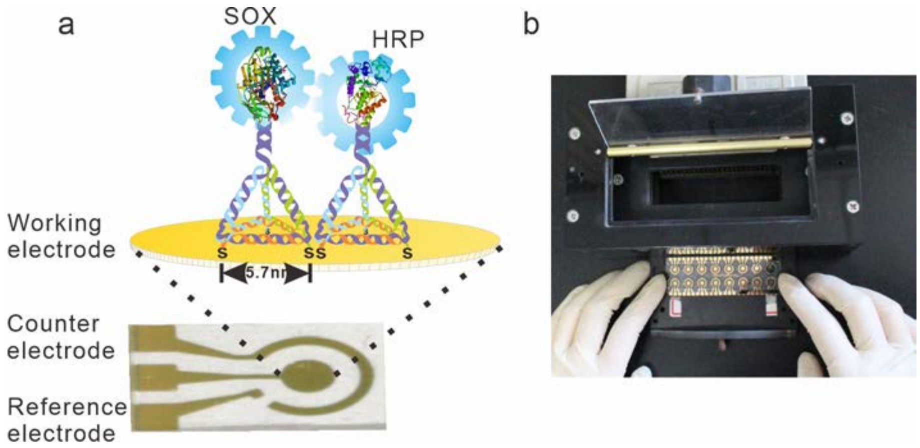

Supplementary Figure 50. Multiplexed electrochemical detection. (a) Electrochemical chip with integrated three-electrode system was employed. Three- electrode system included reference electrode, counter electrode, and working electrode for enzyme immobilization. (b) The electrochemical chip can be employed to multiplexed detection of sarcosine. The chip can be placed into a holder for simultaneous, multiplexed data acquisition.

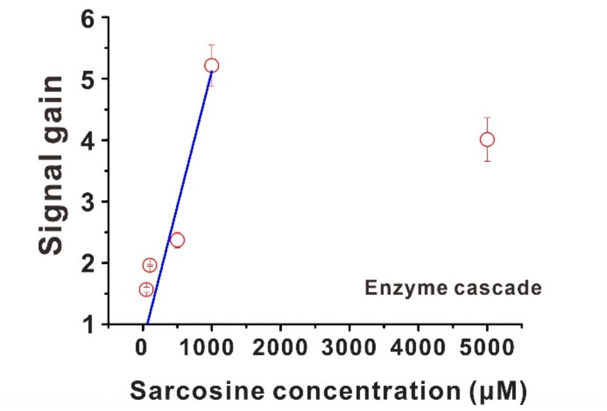

Supplementary Figure 51. The titration curve of sarcosine detection using SOX-HRP cascade reaction in solution. Error bars represent the mean ± s.d. of triplicates. The source data are provided as a Source Data file.

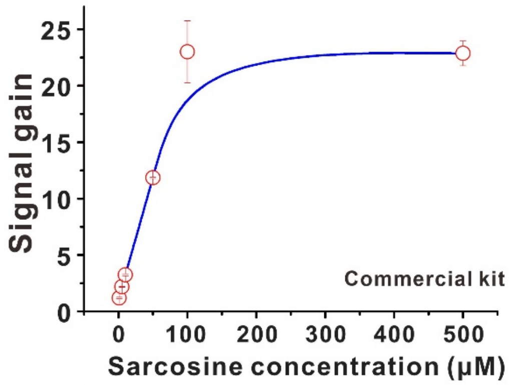

Supplementary Figure 52. The titration curve of sarcosine detection using commercial kit. Error bars represent the mean ± s.d. of triplicates. The source data are provided as a Source Data file.

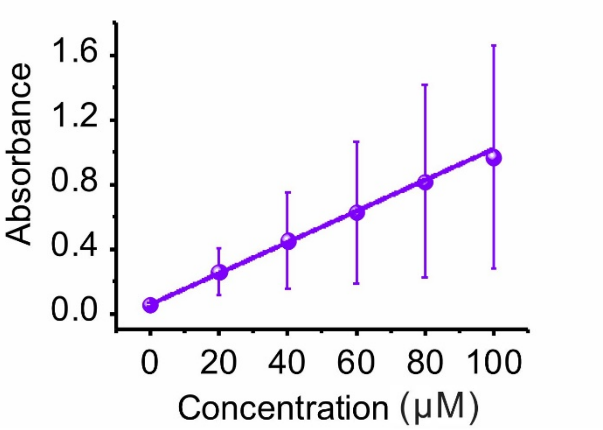

Supplementary Figure 53. Dose-response curves of commercial detection kit for the detection of sarcosine that spiked in in serum. Error bars represent the mean ± s.d. of bipartite. The source data are provided as a Source Data file.

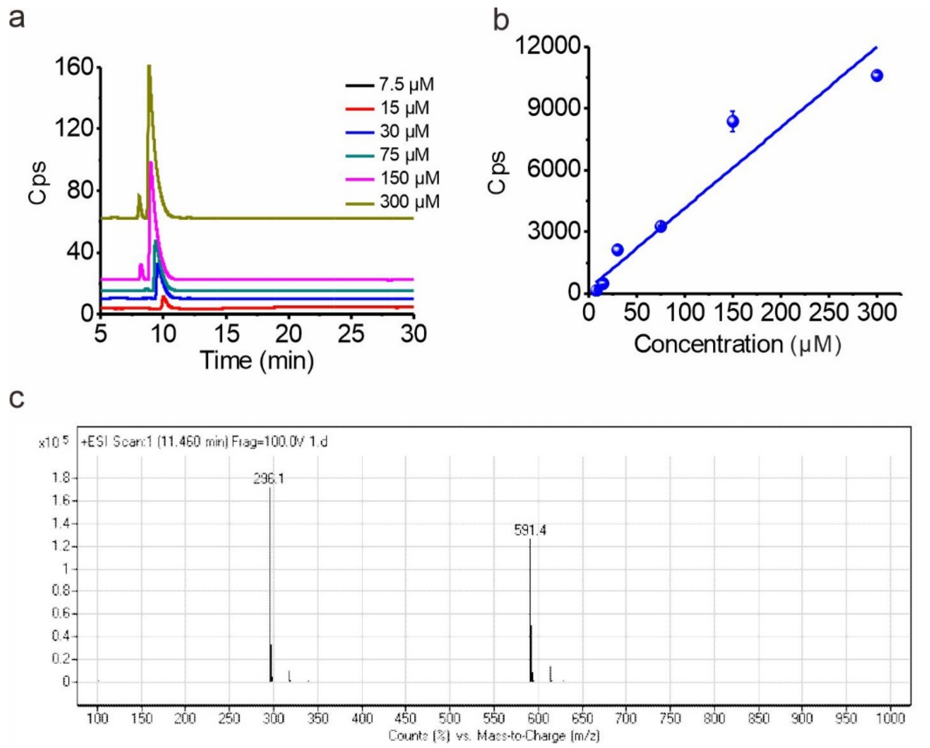

Supplementary Figure 54. Sarcosine quantification by HPLC-MS method after the derivatization step. (a) Cps-time monitoring of sarcosine of different concentration. (b) Linear relation between Cps and sarcosine concentration. (c) MS spectra of sarcosine. Error bars represent the mean ± s.d. of bipartite. The source data are provided as a Source Data file.

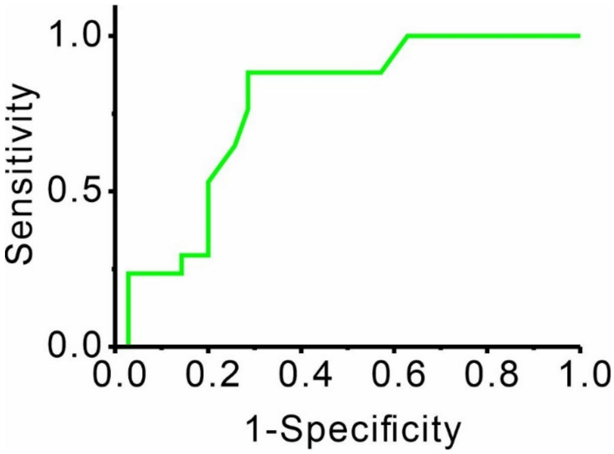

Supplementary Figure 55. The Receiver-operating characteristic (ROC) curves for commercial detection kit. The curve illustrates changes in sensitivity and specificity depending on cutoff. The sample size used to derive statistics is 52. The source data are provided as a Source Data file.

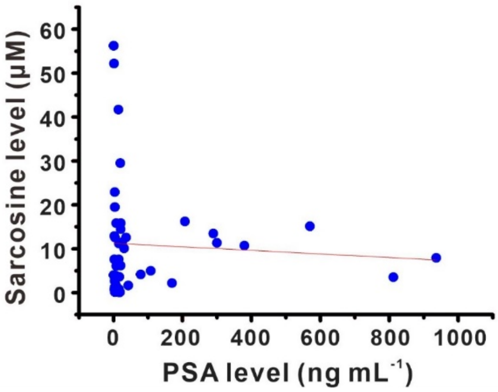

Supplementary Figure 56. Correlation analysis of data from sarcosine detection and PSA detection. The correlation analysis indicated the orthogonality property of these two types of biomarkers. The sample size used to derive statistics is 89. The source data are provided as a Source Data file.

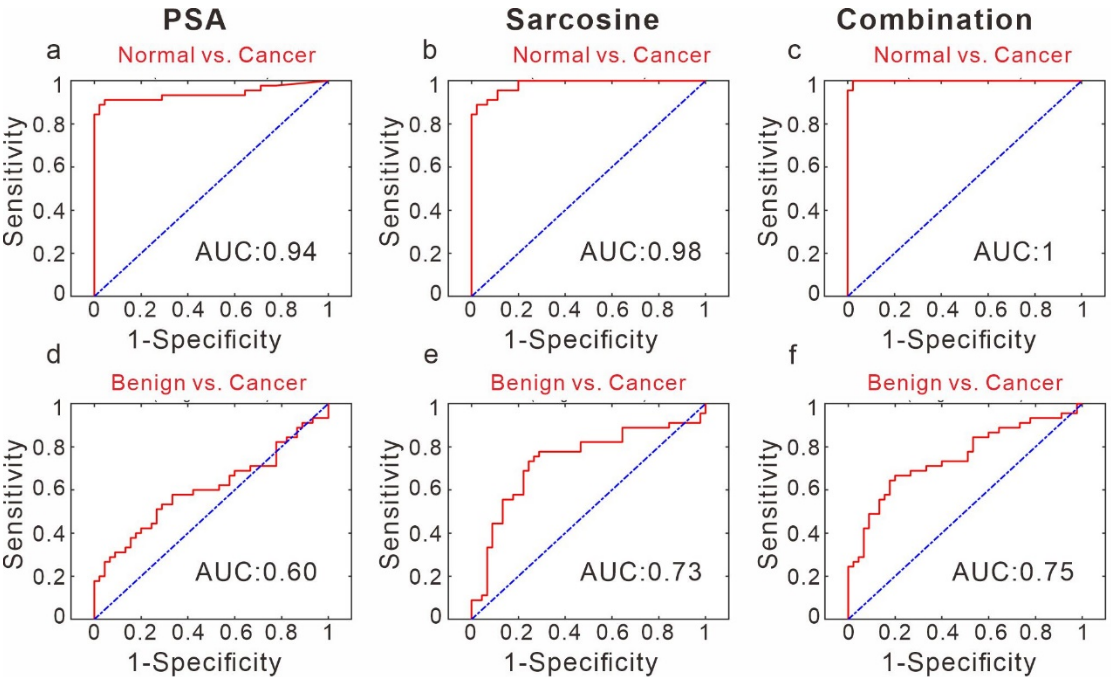

Supplementary Figure 57. ROC analysis for clinical application. ROC analysis for for the discrimination between normal and PCa is based on PSA detection (a), sarcosine detection (b), and the combination of PSA and sarcosine (c). ROC analysis for the discrimination between BPH and PCa based on PSA detection (d), sarcosine detection (e), and the combination of PSA and sarcosine (f). The sample size used to derive statistics is 135. The source data are provided as a Source Data file.

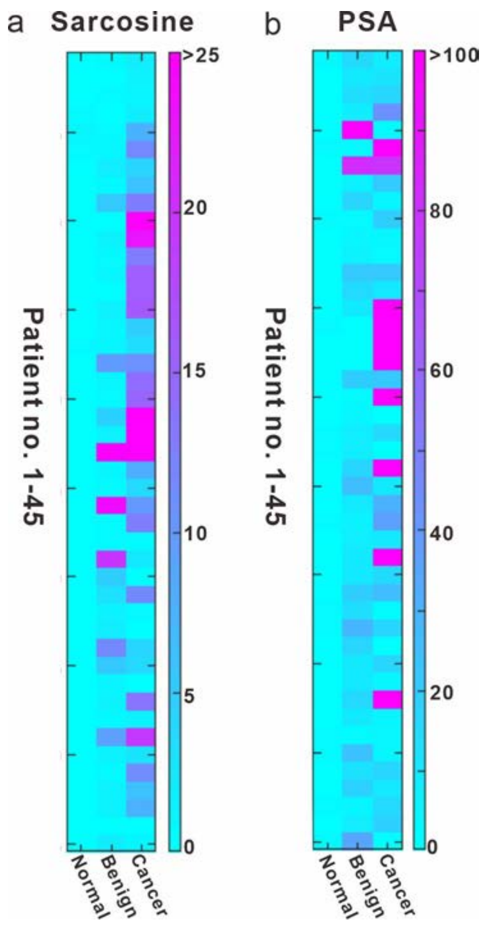

Supplementary Figure 58. The heatmap of sarcosine detection and PSA detection. The heatmap for (a) sarcosine and (b) PSA detection contains the normal controls (45 cases), benign groups (45 cases) and cancer patients (45 cases).

Supplementary Tables

Supplementary Table 1. DNA strands for TDN fabrication

TDN name	DNA name					
17TDN-1	17A$_1$	17B	17C	17D		
17TDN-2	17A$_2$	17B	17C	17D		
17TDN-3	17A$_3$	17B	17C	17D		
17TDN-4	17A$_4$	17B	17C	17D		
17/37TDN-1	17/37-A	17/37-B	37C$_1$	37C$_2$	17/37-D$_1$	17/37-D$_2$-1
17/37TDN-2	17/37-A	17/37-B	37C$_1$	37C$_2$	17/37-D$_1$	17/37-D$_2$-2
37TDN-1	37A$_1$	37A$_2$	37B$_1$	37B$_2$	37C$_1$	37C$_2$
	37D$_1$	37D$_2$-1				
37TDN-2	37A$_1$	37A$_2$	37B$_1$	37B$_2$	37C$_1$	37C$_2$
	37D$_1$	37D$_2$-2				

Supplementary Notes

To explore the CCL-dependence of enzyme cascade at the single-molecule level, we first employed a two-dimensional rectangular DNA origami as nanoruler to program the inter-enzyme distance of an enzyme pair, sarcosine oxidase (SOX) and horseradish peroxidase (HRP). We synthesized a rectangular DNA origami with a ~7000-base circular single stranded M13 DNA and 216 staple strands (Supplementary Table 3). The finite size of the rectangular DNA origami was designed to ~100 ×70 nm (Supplementary Figure 13-18). This rectangular DNA origami as nanoruler has the inherently precise addressability for anchoring biomolecules at nanoscale resolution and single molecule level. We then used this rectangular DNA origami with UPL strands on the surface as template to direct the spatially anchoring of SOX and HRP (Four UPL strands for each enzyme binding site). SOX-DNA (LK 167) or HRP-DNA (LK-191) conjugates (Supplementary Figure 19, 20) were precisely anchored on the surface of DNA origami by hybridizing them to the corresponding complementary UPL strands. The inter–enzyme distance of SOX and HRP can thus be precisely regulated by tuning the site of UPL DNA on the rectangular DNA origami.

Next, we characterized the inter-enzyme distance between SOX and HRP using a combination of single-molecule technique including atomic force microscopy (AFM), total internal reflection fluorescence (TIRF) and super-resolution stochastic optical reconstruction (STORM) microscopy. The distance between SOX and HRP was varied from 10 nm to 70 nm by prescribing UPLs on the origami (10 nm, 30 nm, 50 nm and 70 nm, Supplementary Figure 13-18). The AFM images visualized the successful anchoring of enzyme pair on rectangular DNA origami with programmably controlled inter-enzyme distance (Supplementary Figure 21, 22). We obtained high yield (79% to 88%) for complexes with larger inter-enzyme distance (30 nm, 50 nm and 70 nm) (Supplementary Figure 23-26). For complex with shorter inter-enzyme distance, a lower, yet reasonably good, yield of ~50% was obtained due to the presence of steric hindrance. To identify the coordinately anchoring of SOX and HRP on a single rectangular DNA origami, we performed co-localization analysis by using fluorophore-modified enzymes (Cy3 for SOX, and Alex647 for HRP) and a dual-channel TIRF microscopy (Supplementary

Figure 27-29). The co-localization studies confirmed the simultaneous presence of both SOX and HRP on a single DNA origami. To measure the inter-enzyme distance of SOX and HRP on DNA origami, we employed the STORM microscopy that can image molecules beyond the optical diffraction limit (~200 nm). We established that the inter-enzyme distance were consistent with those in our design (Supplementary Figure 29-31). Of note, the SOX-HRP pair within the 10 nm distance was not resolvable under STORM due to its practical resolution limit (~10-20 nm).

We next examined the CCL-dependent cascade efficiency of the origami-confined enzyme pair. In a typical SOX-HRP cascade reaction, SOX catalyzes the oxidative demethylation of sarcosine, which produces hydrogen peroxide (H_2O_2) that forms the basis for HRP-mediated signal transduction. The enzyme cascade was visualized by the peroxidase-coupled color reaction with a chromogenic substrate-TMB (3,3′,5,5′-Tetramethylbenzidine) (Supplementary Figure 32-33). Interestingly, we find that the reaction velocity was inversely proportional to the inter-enzyme distance of SOX and HRP (Supplementary Figure 34). By taking the yield of the origami-enzyme complex into consideration, we estimated that the SOX-HRP pair within the 10-nm distance exhibited ~15-fold enhancement in reaction velocity as compared to free enzymes in solution (Supplementary Figure 34).

In STORM experiments (Supplementary Figure 29) our STORM imaging and further inter-enzyme distance measurement method was according to the reported method[1] with detailed theoretical and experimental analysis. In briefly, we used fluorescent beads as fiducial markers and obtained over thousands of time-lapse frames. After peak height filtering, drift correction and other correction processes, the gaussian rendering size of our measurement was improved to below 16 nm, which makes the localization precision enough to identify the inter-enzyme distance of 30-70 nm.

For the final clinical application (Supplementary Figures 55-58) the serum samples were collected by the Renji Hospital, Shanghai Jiao Tong University School of Medicine. We have informed consent from the patients. The sarcosine should be stable enough to be stored at –80 °C for at least 4 months, avoiding multiple freeze-thaw processing cycles according to previous studies on the stability of the serum metabolite[2, 3].

Supplementary References

1. Mortensen K. I., Churchman L. S., Spudich J. A., Flyvbjerg H. Optimized localization analysis for single-molecule tracking and super-resolution microscopy. *Nat. Methods* **7**, 377-381 (2010).

2. Breier M., *et al.* Targeted Metabolomics Identifies Reliable and Stable Metabolites in Human Serum and Plasma Samples. *PLoS One* **9**, e89728 (2014).

3. Floegel A., *et al.* Reliability of Serum Metabolite Concentrations over a 4-Month Period Using a Targeted Metabolomic Approach. *PLoS One* **6**, e21103 (2011).
