# Supplementary material for: Programming bulk enzyme heterojunctions for biosensor development with tetrahedral DNA framework
Source: Nat Commun. 2020 Feb 11;11:838. doi: 10.1038/s41467-020-14664-8 (PMC7012893; doi:10.1038/s41467-020-14664-8)
Supplement: Supplementary file 3 — Description of Additional Supplementary Files [file 41467_2020_14664_MOESM3_ESM.pdf]

Description of Additional Supplementary Files

File Name: Supplementary Data 1
Description: DNA sequences for TDN and TDN-enzyme conjugation.

File Name: Supplementary Data 2
Description: DNA sequences for origami and origami-enzyme conjugation

File Name: Supplementary Data 3
Description: The disease Grade of PCa patients.
