# Supplementary material for: Programming bulk enzyme heterojunctions for biosensor development with tetrahedral DNA framework
Source: Nat Commun. 2020 Feb 11;11:838. doi: 10.1038/s41467-020-14664-8 (PMC7012893; doi:10.1038/s41467-020-14664-8)
Supplement: Supplementary file 2 — Peer Review File [file 41467_2020_14664_MOESM2_ESM.pdf]

Editorial Note: This manuscript has been previously reviewed at another journal that is not operating a transparent peer review scheme. This document only contains reviewer comments and rebuttal letters for versions considered at Nature Communications.

REVIEWERS' COMMENTS:

Reviewer #5 (Remarks to the Author):

The authors have clarified my previous queries regarding the source and ethical approval of the patient samples used in the study. The stability of sarcosine was addressed by literature references; given that the stated stability is approximately 4 months, it would be helpful for the authors to indicate the storage time of the samples used in the study.

POINT-BY-POINT RESPONSE

Reviewer #5 (Remarks to the Author): The authors have clarified my previous queries regarding the source and ethical approval of the patient samples used in the study. The stability of sarcosine was addressed by literature references; given that the stated stability is approximately 4 months, it would be helpful for the authors to indicate the storage time of the samples used in the study.

Response: Thanks for the comments. We have indicated the storage time of the samples used in the study. Please see the "Electrochemical Measurements" section in methods in our revised manuscript. "The patients' samples were detected within 4 months after collection.
